# *Terfezia claveryi MAT* locus characterization uncovers evolutionary insights about sexual reproduction of Pezizomycetes and reveals mating type dynamics in mycorrhizal plants

**DOI:** 10.1007/s00572-026-01266-3

**Published:** 2026-05-07

**Authors:** Laura Andreu-Ardil, Ángel L. Guarnizo, Alfonso Navarro-Ródenas, Francisco Arenas, Manuela Pérez-Gilabert, José Eduardo Marqués-Gálvez, Francesco Paolocci, Asunción Morte

**Affiliations:** 1 Departamento de Biología Vegetal (Botánica), Facultad de Biología, Universidad de Murcia, CEIR Campus Mare Nostrum (CMN), Campus de Espinardo, Murcia, 30100 Spain; 2 Departamento de Bioquímica y Biología Molecular-A, Universidad de Murcia, Campus de Espinardo, Murcia, 30100 Spain; 3 Istituto di Bioscienze e Biorisorse, CNR-IBBR, UOS di Perugia, Perugia, 06128 Italy

**Keywords:** Ascomycota, Desert truffle, Mycorrhiza, Arid ecosystems, Heterothallism, Sexual reproductive system

## Abstract

*Terfezia claveryi* is a hypogeous fungus that forms desert truffles through ectendomycorrhizal symbiosis with Cistaceae plants in arid and semiarid environments. The study presented herein elucidates the organization and structure of the mating type (*MAT*) locus in this species and the spatio-temporal dynamics of *T. claveryi* strains in *Helianthemum almeriense* mycorrhizal plants and soil from nursery to field. *MAT* genes are the master loci controlling sexual reproduction and development in fungi. Our findings demonstrate that *T. claveryi* is a haploid and heterothallic species as its strains harbor and express either *TcMAT1-1-1* or *TcMAT1-2-1* genes as revealed by genome sequencing and RNAseq analyses. DNA-binding motifs located in their respective promoter regions appear to play a major role in the regulation of reproductive processes. The α-box and HMG-box domains are highly conserved along the Pezizomycetes and their strong structural similarity despite its poor sequence similarity supports a common evolutionary origin. Moreover, we set out a PCR-based approach to monitor the dynamics of *T. claveryi* strains of opposite mating type on mycorrhizal plants and soil. *T. claveryi* mycorrhizal plants at the nursery stage presented strains of both mating types, whereas a notable dominance of strains with the *TcMAT1-1-1* gene was observed in field stage. Altogether, this research provides insights about genetic regulation and evolution of the *MAT* locus within the Pezizomycetes, and the reproductive biology of this important desert truffle, along with reliable markers to track the spatio-temporal distribution of strains of opposite mating types.

**Supplementary Information:**

The online version contains supplementary material available at 10.1007/s00572-026-01266-3.

## Introduction

*Terfezia claveryi* Chatin is a filamentous fungus belonging to the Pezizaceae family of the Ascomycota phylum, which typically forms ectendomycorrhizal symbiosis with perennial and annual plants of the *Cistaceae* family (e.g. *Helianthemum* spp.) (Gutiérrez et al. 2003; Zitouni-Haouar et al. 2014; Guarnizo et al. 2025). The completion of the sexual reproduction cycle of this fungus leads to the development of edible hypogeous fruiting bodies, commonly referred to as ‘desert truffles’ (Morte et al. 2000). Desert truffles comprise a paraphyletic group of Ascomycota within the Pezizales, including genera such as *Terfezia*, *Tirmania*, *Kalaharituber*, *Mattirolomyces* and *Picoa*. This term arises from their natural distribution in arid and semi-arid regions of the Mediterranean basin, mainly found in Spain, the Middle East, Morocco, Algeria, and Tunisia (Zambonelli et al. 2014). These truffles contribute to rural economic development, especially due to their highly appreciated nutritional and culinary value (Martínez-Tomé et al. 2014; Shavit 2014; Tejedor-Calvo et al. 2021), as well as their antioxidant and antimicrobial properties used in traditional medicine (Shavit and Shavit 2014). Desert truffle sales provide significant sources of income to local communities, support local markets, and contribute to the economic resilience of rural areas across different regions (Alrhmoun et al. 2025; Volpato et al. 2013).

The *Helianthemum almeriense* x *T. clavery*i mutualistic symbiosis plays a crucial role in enhancing plant survival during drought stress by facilitating the exchange of water and key nutrients (N, P, and K) with the host plant (Morte et al. 2000; Andrino et al. 2025). Mycorrhizal associations, such as those of *Cistaceae* plants with desert truffle species, constitute a potential ecological tool not only to prevent desertification in Mediterranean regions, but also to enrich soil fungal and bacterial communities without relying on chemicals (Morte et al. 2009; Berrios et al. 2023). Despite its adaptations, the adverse climatic conditions caused by relentless global warming have resulted in a significant decline in truffle yields, in both natural areas and cultivated plantations (Morte et al. 2021). Thus, understanding the reproductive mode and life cycle of *T. claveryi* is relevant for enhancing desert truffle production in both wild areas and cultivated orchards. Promoting desert truffle yield may in turn facilitate fungal spread within the soil and increase plant colonization levels, thereby helping alleviate the adverse effects of climate change on the symbiotic partners. Despite the considerable ecological and economic repercussions, the life cycle and reproductive biology of the fungi responsible for producing these truffles remain largely unexplored.

Life cycle in Ascomycota initiates once the haploid ascospores germinate. In heterothallic (self-sterile) species, the ascogonium (female partner) and antheridium (male partner) interact in a process called plasmogamy, which leads to the formation of a fruiting body with nuclei of opposite mating types. After karyogamy and meiosis, asci containing a variable number of haploid ascospores are formed. In homothallic (self-fertile) species, the process is similar but does not require a mating partner (Bennett and Turgeon 2016). The regulation of sexual identity in fungal cells is governed by both mating pheromones and the mating-type (*MAT*) locus, commonly known as *MAT1*, since all ascomycetes possess at least one copy of this genomic region (Yoder et al. 1986). The *MAT1* locus harbours two different idiomorphs, *MAT1-1* and *MAT1-2*, which primarily include the *MAT1-1-1* and *MAT1-2-1* genes, respectively (Yoder et al. 1986; Debuchy et al. 2010). These sequences are considered idiomorphic because they occupy the same locus although are highly divergent and non-homologous. In homothallic species, both *MAT1-1* and *MAT1-2* idiomorphs are present within the same haploid genome, eliminating the requirement for mating between genetically distinct types. In contrast, in heterothallic species, each haploid mycelium contains only one of the two idiomorphs, and sexual reproduction requires the combination of complementary idiomorphs from separate individuals (Fraser et al. 2007). Specifically for mycorrhizal heterothallic species, successful reproduction occurs only when (i) strains of opposite mating types interact, allowing the fusion of compatible hyphae and subsequent development of reproductive structures and (ii) at least one of the mating partners uses mycorrhizae to obtain nutrients from the host plants to sustain the growth of developing fruiting bodies (Paolocci et at. 2006; Rubini et al. 2011a; Le Tacon et al. 2013; Wilson et al. 2021). This system promotes genetic diversity by ensuring outcrossing between genetically distinct partners. *MAT1-1-1* and *MAT1-2-1* genes, which encode an α-box domain protein and HMG-box domain protein, respectively, play crucial roles in fungal reproduction, including: (i) acting as master regulators of fertilization by encoding key transcription factors that control sex-related genes, (ii) facilitating mating partner recognition by regulating the release of mating pheromones, and (iii) contributing to the development and maturation of fruiting bodies and ascospores (Bobrowicz et al. 2002; Lee et al. 2003; Kim et al. 2019a).

The genome sequencing of the mycorrhizal strains *T. claveryi* T7 and *Tirmania nivea* G3 suggested that they are heterothallic species, since, in both cases, solely the *MAT1-1-1* DNA sequence was found (Marqués-Gálvez et al. 2021). In order to gain insight into the reproductive mode of desert truffles, the *MAT* locus of *T. claveryi* was studied. For this purpose, we (i) screened *T. claveryi* strains for the presence/absence of the *MAT1-1-1* gene; ii) PCR amplified and sequenced the *MAT1-2-1* gene from a strain lacking the opposite mating type; iii) assessed the evolutionary relationship of both *MAT1-1-1* and *MAT1-2-1* genes with those from other Pezizomycetes; (iv) set a reproducible PCR-based strategy to monitor the spatio-temporal distribution of strains of opposite mating type in soil and on root samples from inoculated *H. almeriense* plants. This work advances our understanding of the reproduction mode of desert truffle fungi and highlights its implication in ecological development of managed ecosystems from nursery to field.

## Materials and methods

### Experimental setup, biological material and sampling

For *MAT* locus characterization, free living mycelium (FLM) grown in vitro on cellophane and placed over agar plates containing optimal MMN medium (Arenas et al. 2018) from two different *T. claveryi* strains (Tc1705 and TcLlano) were used. These strains belong to the Mycology-Mycorrhiza-Plant Biotechnology group collection at University of Murcia and were maintained in vitro in an incubator at 24 °C in the dark. After collection of at least 100 mg of FLM from each strain, the material was placed in Eppendorf tubes, flash frozen in liquid nitrogen and stored at -80 °C until DNA isolation. In addition, for DNA isolation from individual spores, we also sampled an ascocarp of *T. claveryi* collected in 2005 in Zarzadilla de Totana (Murcia, Spain) which was stored at -20 °C until use.

For the assessment of the spatio-temporal distribution of *MAT* genes, we sampled all fine roots from the root system and soils from *H. almeriense* x *T. claveryi* mycorrhizal plants at nursery stage (300 ml pots) and at field stage. A total of 405 *H. almeriense* plants were inoculated with spore inocula from a mix of 2–3 *T. claveryi* ascocarps with mature spores in December 2021, following the protocol described by Morte and Andrino (2014). In total, 71 soil samples and 66 root samples were collected from February 2022 to March 2024 to evaluate the spatio-temporal distribution of *TcMAT1-1-1* and *TcMAT1-2-1* genes (Supplementary Table 1). Root and soil samples were collected (i) in nursery at 2, 4, 6, and 9 months post inoculation (times T2-T9), and (ii) in field at 14, 18, and 27 months post inoculation (times T14-T27). Each sampling-time corresponds to 8–13 individual plants and surrounding soils, which act as 8–13 biological replicates. Nursery plants were transplanted into an experimental plantation located in Cañada de Canara (Murcia, Spain, 38°06’47.3"N, 1°45’56.5"W). The plantation was systematically mapped by establishing ten unique plots and, for each sampling event, one random sampling point per plot was selected to ensure spatial representation (Supplementary Fig. 1). Sample collection in field was carried out using a spade at a depth of 10 cm and at 10–15 cm from the plant base, approximately. Soil and root samples were dried at room temperature and then stored at -20 °C in microcentrifuge tubes until DNA isolation.

### DNA isolation

Different approaches were followed for the isolation of genomic DNA depending on the source of the sample: soil samples, root samples, FLM from in vitro cultured strains or individual spores from the same *T. claveryi* ascus. Three steel beads of 3 mm diameter were added to microcentrifuge tubes containing root, soil or FLM samples, which were then homogenized for 2 min at a frequency of 30 Hz using the TissueLyser II (QIAGEN, Germany) as dry material (soil) or pre-immersed in liquid nitrogen to facilitate plant and fungal cell disruption (roots and FLM). DNA from soil was extracted using the NucleoSpin^®^ Soil kit (Macherey-Nagel, Düren, Germany), following the manufacturer’s instructions with some modifications: 500 µl of the lysis buffer SL1 and 150 µl of the SX enhancer solution were used. DNA from root samples and FLM was extracted using the CTAB method (Chang et al. 1993). Subsequently, all samples were purified using the DNeasy^®^ PowerClean^®^ Pro Cleanup kit (Hilden, Germany), according to the manufacturer’s guidelines. In addition, DNA isolation of individual spores belonging to the same ascus of semi-mature *T. claveryi* gleba (spores with full ornamentation but still inside the asci, Supplementary Fig. 2) was carried out following the methodology reported by Paolocci et al. (2006); Rubini et al. (2011a). The concentration and quality of DNA samples was determined using a NanoDrop ND-2000 Spectrophotometer (Thermo Fisher Scientific, Waltham, MA, USA). These samples were stored at -20 °C until further use.

### Primer design and PCR protocol for the identification of *TcMAT* genes

To corroborate the identity of the *T. claveryi* mycelium strains, PCR methodology using primers targeting the ribosomic DNA (rDNA) region (ITS1F and ITS4) was performed (White et al. 1990), according to Bordallo et al. (2013). Primers targeting the *TcMAT1-1-1* gene, which was previously identified by Marqués-Gálvez et al. (2021), were designed by using the putative sequence of *MAT1-1-1* found in scaffold 39 (genomic coordinates, 66965–70230) of *T. claveryi* available in Mycocosm database (https://mycocosm.jgi.doe.gov/Tercla1/Tercla1.home.html) (Table 1). Using as a template the *T. claveryi* genome, the 453 and 455 primer pair (Table 1), designed on the putative regions flanking the *TcMAT* locus was employed to amplify the strains of the collection. This PCR was carried out in FlexCycler thermal cycler (Analytik Jena, Jena, Germany) in a final volume of 50 µl following a two-step procedure: initial denaturation at 98 °C for 30 s, followed by 35 cycles of (i) denaturation at 98 °C for 7 s, and (ii) annealing and extension at 72 °C for 2 min, with a final extension at 72 °C for 7 min. The resulting amplicons obtained from strains with (Tc1705) or without (TcLlano) the *TcMAT1-1-1* gene as per PCR with *TcMAT1-1-1* specific primer pair were sequenced by Plasmidsaurus (https://plasmidsaurus.com) using Oxford Nanopore Technology with custom analysis and annotation, which allows sequencing of the entire amplicon in a single read. The consensus sequences obtained were used to confirm the specificity of *TcMAT1-1-1* primers and design *TcMAT1-2-1* specific primers. All primers were designed with the help of PerlPrimer (Marshall 2004) and manually fine-tuned afterwards.

**Table 1:** List of primers employed for the optimized PCR-based characterization of *T. claveryi* reproductive genes and *T. claveryi* qPCR-quantification

Primer ID	Sequence (5’◊3’)	Location	Length (nt)	Tm ( °C)	Amplicon (pb)	GC (%)
ITS1F	CTTGGTCATTTAGAGGAAGTAA	18 S rRNA gene	22	58.0	633	36.36
ITS4	TCCTCCGCTTATTGATATGC	28 S rRNA gene	20	61.0		45.00
453	GGTAATTGCGGTCGGGGATTCTGG	*MAT* flanking locus	24	69.0	2868 (*MAT1-1-1*) 3087 (*MAT1-2-1*)	58.33
455	TTCCGCGCACAGTGAGTCCATCATTATT	*MAT* flanking locus	28	70.1		46.43
MAT111BFwd	TGTCTCCACTGTCTCTATCTTTGCTG	*MAT1-1-1*	26	65.8	273	46.15
MAT111BRev	AAGCGTGGTTGAAAGTCGTGTTC	*MAT1-1-1*	23	65.7		47.83
MAT121Fwd	CTCCACCTCTAAGCAACCTTCCA	*MAT1-2-1*	23	65.7	434	52.17
MAT121Rev	GTACTGAATTCCGTTCTGCTTCGAGAT	*MAT1-2-1*	27	66.5		44.44
TerclaF3	GCTCCCCCTCACTCAAGTAT	*ITS rDNA*	20	59.1	79	55
TerclaR1	TGGAGGGCAACTTAATACACAGT	*ITS rDNA*	23	59.2		43

The final optimized procedure for *T. claveryi MAT* gene identification in all soil, root, FLM and spore samples consisted of a nested PCR reaction containing 0.2 mM of each dNTP, 0.2 µM of each primer, 2.5–50 ng of gDNA, 1X of Phusion™ HF Buffer and 0.02 U/µl of Phusion™ High - Fidelity DNA Polymerase (Thermo Fisher Scientific Baltics UAB, Lithuania) (Supplementary method 1). The first PCR employed the 453–455 primer pair and was carried out as explained above. After this reaction, PCR products were purified using the GeneJET PCR Purification Kit (Thermo Fisher Scientific Baltics UAB, Lithuania) according to the manufacturer’s guidelines. The second PCR reaction was performed in a 20 µl volume using the previous purified PCR product as template with MAT111BFwd-MAT111BRev and MAT121Fwd-MAT121Rev specific primers, following a three-step program: initial denaturation at 98 °C for 30 s, followed by 35 cycles of (i) denaturation at 98 °C for 7 s, (ii) annealing at 66 °C for 10 s, and (iii) extension at 72 °C for 30 s, with a final extension at 72 °C for 7 min. For the second reaction, a multiplex PCR including both *MAT1-1-1* and *MAT1-2-1* specific primer pairs, was applied to the analysed samples. Each nested PCR experiment included (i) two non-DNA-template negative control, one from the first PCR and another for the second PCR, and (ii) positive controls: DNA from a strain of *TcMAT1-1-1*, another of *TcMAT1-2-1*, and a 1:1 (v: v) mix of both. Final PCR products were loaded and separated on an agarose gel stained with SYBR™ Safe DNA Gel Stain (Thermo Fisher Scientific, Life Sciences Solutions, CA, USA) and photographed under an UV transilluminator (MultiImage™ Light Cabinet, Alpha Innotech, San Leandro, CA, USA). This protocol was applied to all samples to characterize the presence of *T. claveryi* MAT genes (Supplementary Table 1). Moreover, the nested PCR was carried out on serially diluted (1:10, 1:100; 1:1000; 1:10000) FLM DNA of each strain to assess the detection limit of both primer pairs. In addition, two putative *TcMAT1-1-1* and *TcMAT1-2-1* amplicons, each one obtained by conventional nested PCR from a different spore of the same ascus, were sequenced using Sanger sequencing (3500 Genetic Analyzer, Applied Biosystems, Waltham, MA, USA) at the Molecular Biology Service belonging to the “Area Científica y Técnica de Investigación” (ACTI, Universidad de Murcia) and aligned to the MAT1-1-1 and MAT1-2-1 sequences.

### Genomic structure prediction of *T. claveryiMAT* locus

Using the previously sequenced *MAT* locus, in silico gene structure prediction of *TcMAT1-1-1* and *TcMAT1-2-1* was performed with FGENESH software (Solovyev et al. 2006, http://www.softberry.com/berry.phtml) using *Neurospora crassa* as a reference genome. Additionally, RNA-seq raw reads from FLM, well-watered and drought-stressed *H. almeriense* x *T. claveryi* mycorrhizal roots (Short Read Archive ID: SRP272077, Marqués-Gálvez et al. 2021) were used to validate the predicted gene structure at experimental level. The reads were trimmed using TRIMMOMATIC (v0.39, Bolger et al. 2014) and mapped against the previously sequenced *TcMAT1-1-1* and *TcMAT1-2-1* concatenated amplicons, using HISAT2 (v2.2.1, Kim et al. 2019b). Mapped reads were visualized using IGV viewer (v2.11.9, Robinson et al. 2011). Once gene structure was defined for both *TcMAT1-1-1* and *TcMAT1-2-1* idiomorphs, promoter sequences were defined as the non-coding sequence upstream of the transcription starting site (TSS) of each gene. DNA-binding motifs were searched by analysing these sequences with Find Individual Motif Occurences (FIMO, v5.5.8, Grant et al. 2011) online tool, using the non-redundant JASPAR 2024 CORE database for fungi (https://jaspar2024.elixir.no/). The presence of transposable elements in the *TcMAT* idiomorphs was studied using the GIRI database tool (https://www.girinst.org).

### Protein domain recognition, structural alignment and phylogenetic analysis

The *TcMAT1-1-1* and *TcMAT1-2-1* sequences were analysed using the InterPro database and its domain prediction tools (Blum et al. 2024). The three-dimensional structures of α- and HMG-box domains encoded by these genes were predicted using the AlphaFold Server, which utilizes the AlphaFold 3 model (Abramson et al. 2024). Default parameters were applied, incorporating geometric constraints through the structure refinement. The resulting structural models were evaluated using MolProbity (Williams et al. 2018) to assess stereochemical quality and all-atom contacts. For each predicted protein, MolProbity scores and validation metrics, including clashscore, Ramachandran plot statistics, rotamer outliers, bond and angle geometry, and Cβ deviations, were calculated. The model displaying the most favourable validation metrics was selected for further structural analysis. Structural superposition of the secondary structures of the conserved domains was performed using UCSF Chimera v1.19 (Pettersen et al. 2004). Structural alignment was conducted with the Matchmaker tool, excluding long atom pairings iteratively until no aligned atom pairs exceeded a distance threshold of 2.0 Å and ensuring accurate and meaningful structural overlap.

Two phylogenetic trees were constructed based on the α- and HMG-box domains encoded by orthologous MAT genes within the Pezizomycetes. Protein sequences were selected according to their phylogenetic proximity and obtained from the JGI MycoCosm database (https://genome.jgi.doe.gov/mycocosm/home). For the α-box domain analysis, 8 orthologous sequences were selected, whereas 11 orthologs were used for the HMG domain (Supplementary Table 2). In addition, two phylogenetic trees using the ITS sequences of the studied species were also constructed. *Naumovozyma castellii* and *Rhizopus stolonifer* were included as outgroups for the α- and HMG-box domain trees, respectively. Multiple sequence alignments were performed using MUSCLE implemented in MEGA v11 (Tamura et al. 2021). Model selection was carried out using 8 computational threads, while all other parameters were kept at default settings. Phylogenetic trees were inferred using the Maximum Likelihood method with 1,000 bootstrap replicates to assess branch support. The LG + I and T92 + G + I models were identified as the best-fit evolutionary models and were applied to both the α- and HMG-box domains datasets, and the ITS trees, respectively.

CDS nucleotide sequences of the selected *MAT1-1-1* and *MAT1-2-1* genes of different Pezizomycetes fungi (Supplementary Table 2) were aligned using TranslatorX (Abascal et al. 2010), which performs codon alignments by translating DNA sequences into amino acids, aligning them at the protein level, and then back-translating to nucleotides. The alignment was generated using the MUSCLE algorithm for protein alignment, and poorly aligned regions were removed using Gblocks. The resulting clean nucleotide alignment was selected for further analyses, as it maintains codon alignment while excluding unreliable sites. To detect signals of selection, the Fixed Effects Likelihood (FEL) method was applied using the Datamonkey web server (Weaver et al. 2018), under the assumption of pervasive selection acting uniformly at each codon site across the phylogeny (Kosakovsky Pond and Frost 2005).

### *T. claveryi* quantification

The amount of *T. claveryi* DNA in all soil and root samples, which previously were analysed for *TcMAT* genes detection, was determined by qPCR methodology using a QuantStudio™ 5 Flex instrument (Applied Biosystems, Waltham, MA, USA) (Supplementary Table 1). A standard curve was generated from 1:10 dilutions of purified ascocarp DNA to assess the efficiency of the primers. The total volume of each qPCR reaction was 10 µl, consisting of 1X PowerTrack™ SYBR™ Green Master Mix (Thermo Fisher Scientific Baltics UAB, Lithuania), 0.3 µM of each primer (TerclaF3 and TerclaR1, designed by Arenas et al. (2022a), and 30–45 ng of gDNA. The thermal cycling conditions were: 50 °C for 2 min, 95 °C for 2:30 min (for enzymatic activation), followed by 40 cycles of 95 °C for 15 s and 60 °C for 1 min. Three technical replicates of each sample and standard were performed, and a non-DNA-template was included in each run to ensure the absence of cross-contamination.

### Statistical analysis

Statistical analyses were conducted in R v.4.3.3 (Posit team 2024). Linear models (LMs) were fitted to assess the effects of experimental factors on the abundance of *T. claveryi*. The response variable was transformed to the square root of ng/mg of sample to improve normality and variance homogeneity. Pairwise comparisons among factor levels (source, time, mating type and location) were performed using estimated marginal means (EMMs) computed with the emmeans package (Lenth 2025). For the combination of variables source and location, pairwise comparisons were conducted applying Bonferroni correction. In all other models, Tukey’s HSD adjustment was applied for multiple comparisons. A significance threshold of *p* ≤ 0.05 was used throughout the analyses. Data visualization was performed using the ggplot2 package (Wickham 2016).

## RESULTS

### PCR protocol optimization and determination of the sexual mating system of *Terfezia claveryi*

**Fig. 1:**
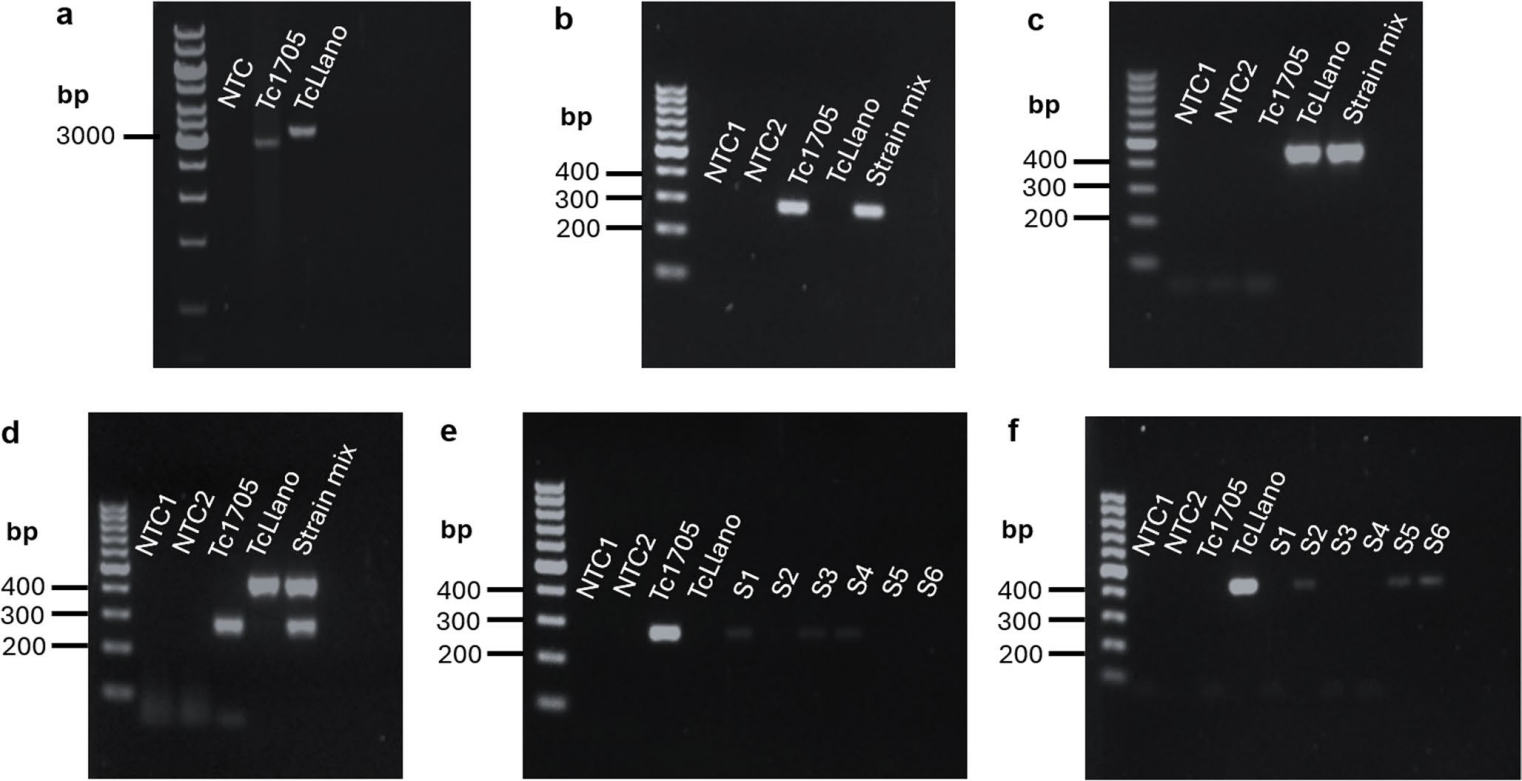
Genetic evidence of *T. claveryi*’s heterothallism. Agarose gel electrophoresis showing PCR products obtained from: (**a**) a direct reaction carrying 453–455 primer pairs; a nested reaction using (**b**) MAT111BFwd-MAT111BRev primers and (**c**) MAT121Fwd-MAT121Rev primers; and (**d**-**f**) a nested multiplex PCR using MAT111BFwd-MAT111BRev and MAT121Fwd- MAT121Rev. Strain mix: Tc1705:TcLlano (1:1); S1-S6: individual spores isolated from the same ascus. Uncropped images can be consulted in Supplementary Dataset 1

To experimentally determine the mating system of *T. claveryi*, we first screened FLM strains by PCR using both rDNA and MAT1-1-1 primers. Tc1705 and TcLlano showed 98% and 99% of similarity, respectively, with the described ITS sequences of *T. claveryi*. Samples that produced the expected amplicons were identified as *TcMAT1-1-1* carrier strains. The TcLlano strain, which tested positive for ITS amplification but negative for amplification with *TcMAT1-1-1* specific primer pair, was then selected as a putative strain carrying the *TcMAT1-2-1* gene. Subsequently, a PCR to amplify the putative *MAT* idiomorphs from strains harbouring (Tc1705) or lacking (TcLlano) the *TcMAT1-1-1* gene was performed by using the primers 453–455 designed on putatively conserved regions flanking the *MAT* locus (Marqués-Gálvez et al. 2021). Tc1705 and TcLlano strains showed distinct results, which suggested the presence of different idiomorphs. The obtained PCR products sized 2,925 kb for Tc1705 and 3,275 kb for TcLlano (Fig. 1A). The sequencing of these amplicons confirmed genetic polymorphism between the strains. In fact, the sequence from Tc1705 aligned with the putative *MAT1-1-1* of *T. claveryi* (81% of identity with scaffold 39, genomic coordinates: 66965–70230) (Supplementary Data), whereas TcLlano aligned with putative *MAT1-2-1* of *T. boudieri* (79% of identity with scaffold 37, genomic coordinates: 129511–135835) (Supplementary Data).

Using the sequences obtained from the first PCR products, a new primer pair specific to *TcMAT1-1-1* (MAT111BFwd-MAT111BRev) and one to *TcMAT1-2-1* (a MAT121Fwd-MAT121Rev) were designed and used to amplify the 453–455 PCR products obtained from both Tc1705 and TcLlano strains. When this second PCR was performed as a non-multiplexed PCR, Tc1705 produced a 270 bp band with MAT111BFwd-MAT111BRev primer pair, but none with MAT121Fwd-MAT121Rev primers, as expected (Fig. 1B). The opposite result was found for TcLlano, which amplified a 430 bp band with MAT121Fwd-MAT121Rev primer pair but not with MAT111BFwd-MAT111BRev (Fig. 1C). When this second reaction was performed as a multiplex PCR, including both primer pairs in the same reaction, again Tc1705 strain produced a single 270 bp amplicon, while TcLlano strain produced a single 430 bp amplicon. Additionally, when both templates were mixed at a 1:1 proportion, two amplicons (270 and 410 bps) were produced concomitantly. Overall, these results indicate that Tc1705 harbours the *MAT1-1-1* gene, whereas TcLlano is a *MAT1-2-1* strain carrier (Fig. 1D).

In order to provide another line of evidence of the heterothallic nature of *T. claveryi*, we isolated DNA from six individual spores from the same ascocarp and performed the same nested PCR protocol. In this case, the second reaction was performed as a non-mutiplex, since multiplex results were not consistent. Six individual spores derived from a single ascus of semi-mature gleba exhibited a consistent pattern, in which three of them tested positive solely for *MAT1-2-1* and the remaining three were positive only for *MAT1-1-1* (Fig. 1E-F), thereby reinforcing the concept not only of heterothallism but also of the haploid nature of the *T. claveryi* spores. The sequencing of PCR products from two distinct spores belonging the same ascus, one positive for *MAT1-1-1* and the other for *MAT1-2-1*, revealed identical sequences to those obtained from amplicons of pure mycelium Tc1705 and TcLlano strains. Overall, the results from the molecular approach provided strong evidence for the occurrence of two distinct, non-concurrent idiomorphs at the *MAT* locus in *T. claveryi*, indicating its heterothallic nature.

### Characterization of the *MAT* locus of *T. claveryi*

**Fig. 2:**
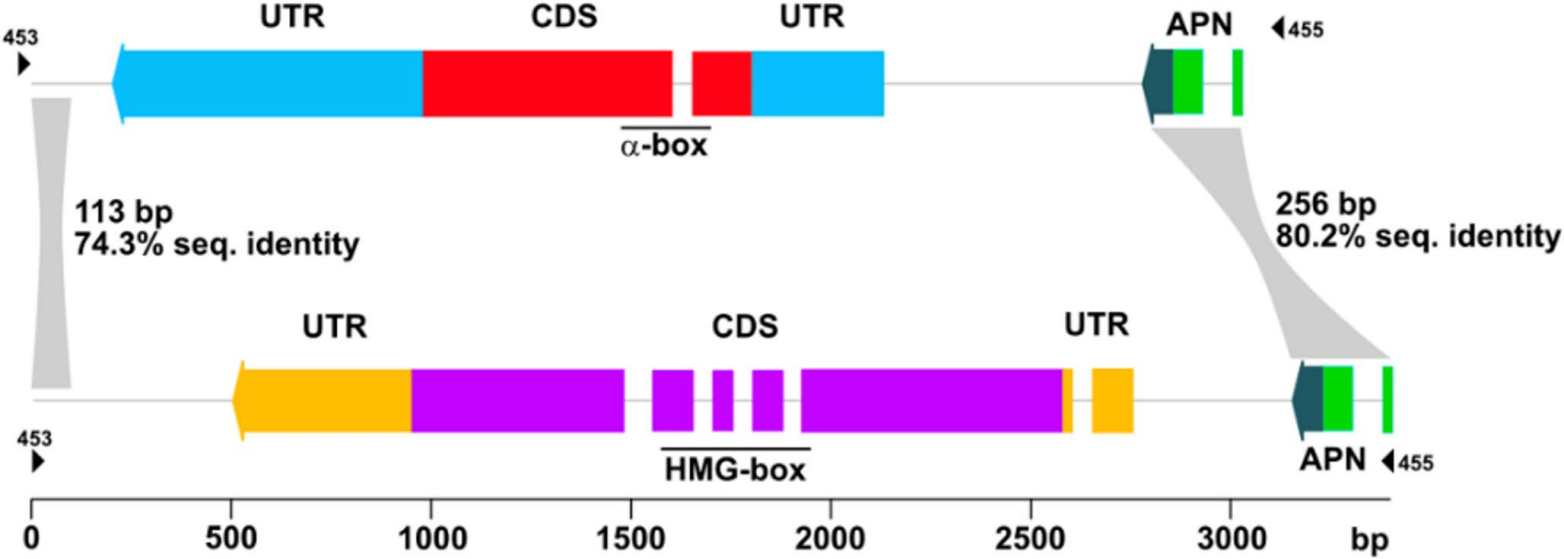
Schematic representation of the *T. claveryi* mating type locus. Sequences amplified by 453 and 455 primers, showing the idiomorphic regions *TcMAT1-1* (top) and *TcMAT1-2* (bottom). The *MAT1-1-1* gene is represented as a red box and contains an α-box domain, whereas the *MAT1-2-1* gene is shown as a purple structure containing an HMG-box domain. Untranslated regions (UTRs) are represented in blue for *TcMAT1-1-1* and orange for *TcMAT1-2-1*. Both idiomorphs are flanked by a conserved Apurinic/apyrimidinic endonuclease (APN) gene (green box). Two homologous regions are shared between idiomorphs, which are represented as grey boxes, with a diagonal grid pattern

Both the in silico gene structure prediction based on the sequenced *MAT* amplicons (Supplementary Figs. 3 and 4) as well as RNA-seq data from *H. almeriense x T. claveryi* mycorrhizal roots (Supplementary Fig. 5) revealed the same gene length and genomic organization of the *MAT* locus, including the same intron number and positions in both *TcMAT* genes (Fig. 2).

Both sequences were oriented in the antisense direction, relative to the reference genome. *TcMAT1-1-1* (2,868 pb) showed a slightly smaller DNA sequence than *TcMAT1-2-1* (3,087 pb). In addition, the coding region of *TcMAT1-1-1* contained a single intron located within the α-box domain, whereas the coding region of *TcMAT1-2-1* exhibited a more complex exon-intron structure since it contained five introns, three of which were in the HMG-box domain. Another intron was found within the CDS, while the last one was located at the 5′ UTR region in *TcMAT1-2-1*. The regions flanking the idiomorphs were highly conserved, with 74.3% sequence identity in the 113 bp 5′ segment and 80.2% sequence identity in the 256 bp 3′ segment, which included the coding region of an apurinic/apyrimidinic endonuclease (APN). The CDS sequence of *TcMAT1-1-1* encoded for a smaller protein (254 aa) than that encoded by *TcMAT1-2-1* (446 aa). An 812 bp promotor region was identified between *APN* and *TcMAT1-1-1* genes, whereas it was of 504 bp for *TcMAT1-2-1*. We discovered several DNA-binding motifs for both promoter regions (Table 2), including (among others) three copies of the CHA4 motif (GGCGGAGA) and three copies of the IME1 motif (CGGCGGAG) in *TcMAT1-1-1*_*pro*_, two copies of the EDS1 motif (GGAAAAA) in *TcMAT1-2-1*_*pro*_ and YPR196W motif ([G/A][A/T]TTC[T/A]CCG), which was found in both promoter regions. No transposable elements were found in either idiomorph.

**Table 2:** DNA-binging motifs identified in *TcMAT1-1-1 *and *TcMAT1-2-1 *promoters. Motifs were identified by searching the 812 bp and 504 bp promoter sequences of *TcMAT1-1-1* and *TcMAT1-2-1*, respectively, using FIMO tool (see materials and methods). Only those hits with positive score and statistical significance (*p* ≤ 0.01) are shown

Promotor region	TF (Motif ID)	Motif sequence	Position*	Strand	Score	*p*-value	Known biological role
*TcMAT1-1-1*	CHA4 (MA0283.1)	GGCGGAGA	413 390 344	+	14.1463	9.90e-06	Involved in aminoacidic metabolism. Responsible of the use of serine/threonine as nitrogen sources (Holmberg and Scherling, 1996)
	STB3 (MA 0390.2)	ATTTTT TCATG	624	-	13.6557	1.22e-05	Regulator of ribosome biogenesis genes under nutrient stress. Repression of growth under quiescence (Liko et al. 2010)
	SWI4 (MA0401.1)	ACGCGAAA	135	-	14.3293	1.47e-05	Involved in the regulation of transcription of cell cycle-dependent genes (Baetz and Andrews 1999)
	IME1 (MA0320.1)	CGGCGGAG	412 389 343	+	12.6571	3.25e-05	*Inducer of meiosis 1* (*IME1*) encodes a transcription factor required for sporulation and meiosis processes (Mandel et al. 1994; Chu and Herskowitz 1998)
	ZMS1 (MA0441.2)	CCCCGCA	389	+	12.6098	3.62e-05	Regulator of genes related to glycerol-based growth and cellular respiration (Lu et al. 2005)
	MAC1 (MA0326.1)	TGTGCTCG	668	-	11.7586	5.93e-05	Copper-fist DNA binding domain. Regulation of the Cu/Fe utilization and stress resistance (Yonkovich et al. 2002)
	SUM1 (MA0398.2)	TAATTTTT	626	-	10.619	6.46e-05	Related to repression of sporulation genes during vegetative growth through the recruitment of a histone deacetylase (Xie et al. 1999)
	TBF1 (MA0403.3)	AACCCTGA	89	+	11.6053	7.98e-05	TBF1 encodes a protein that binds to telomeric TTAGGG repeats, regulates telomere length, and controls gene expression by acting as a chromatin insulator (Koering et al. 2000)
	NDT80 (MA0343.2)	GACACAAAC	434	+	10.4404	9.09e-05	NDT80 encodes a transcription factor which is involved in the activation of genes for meiosis and spore formation, competing with the repressor SUM1 (Pierce et al. 2003)
	YPR196W (MA0437.1)	GATTCTCCG	532	+	11.387	6.6e-05	C2H2 zinc finger motif. Poorly characterized. Enriched in hexose transporters in yeasts (Badis et al. 2008)
*TcMAT1-2-1*	YPR196W (MA0437.1)	ATTTCACCG	132	-	11.387	6.6e-05	
	IXR1 (MA0323.1)	AAACGGTTGCGGGT	258	-	8.06579	1.11e-05	High mobility group (HMG) related to the regulation of hypoxic genes (Castro-Prego et al. 2010)
	LEU3 (MA0324.1)	CCGGTTGCGG	269	-	13.3659	2.35e-05	The regulatory protein LEU3 (LEUR) controls a group of leucine-specific genes (Sze et al. 1992)
	SWI4 (MA0401.1)	ACGCGAAT	319	+	12.9024	2.94e-05	The SWI4 transcription factor, together with SWI6, forms the SBF complex, responsible for activating genes in the G1/S transition of the cell cycle (Baetz and Andrews 1999)
	MCM1 (MA0331.1)	CCTAATTGGCA	298	-	11.3878	5.65e-05	Involved in the activation of early cell cycle genes (G1/M), regulation of mating-type genes, and coordination of the arginine metabolism genes (Elble and Tye 1991; Messenguy and Dubois 1993)
	HSF1 (MA0319.2)	ATGGAAC	207	+	12.1341	6.51e-05	The heat shock transcription factor (HSF1) activates heat shock proteins (HSPs) to protect cells from stress by maintaining proper protein folding and preventing damage (Wiederrecht et al. 1988)
	EDS1 (MA0294.2)	GGAAAAA	161 44	+	11.878	7.93e-05	Member of C6 zinc cluster factors. Poorly characterized.
	ARR1 (MA0274.1)	ATCTGAAT	354	+	10.9221	8.79e-05	The arsenical-resistance protein (ARR1) is involved in the cellular response to arsenic-containing substances (Menezes et al. 2004)
	ASH1 (MA0276.1)	CCAAATTAGG	309	+	10.7429	9.73e-05	Involved in in chromatin organization, negative regulation of mating-type switching and promotion of pseudohyphal growth (Sil and Herskowitz 1996; Pan and Heitman 2000)
	RME1 (MA0370.1)	TGTAAAGGGA	31	+	10.7237	9.85e-05	Regulator of meiosis, pseudohyphal growth and the G1/S transition. Promoter of invasive growth under glucose limitation (Toone et al. 1995)

*Upstream TSS

### The aminoacidic sequences of the conserved α- and HMG-box domains are highly preserved along different Pezizomycetes fungi

**Fig. 3:**
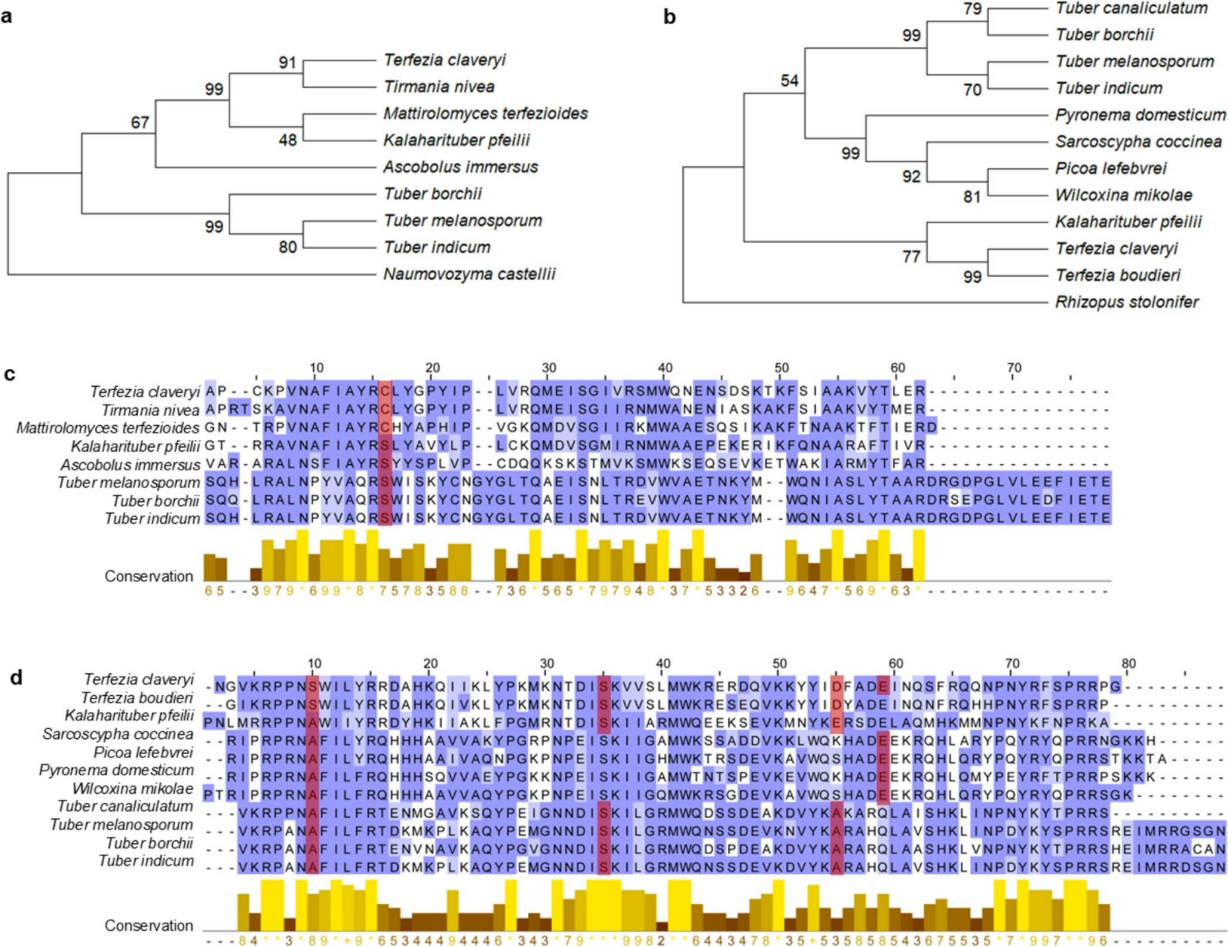
Phylogenetic relationships and alignment of α-box and HMG-containing domains in Pezizomycetes. Maximum likelihood phylogenetic trees inferred from the predicted amino acid sequences of conserved mating-type protein domains: (**a**) α-box domain (encoded by *MAT1-1-1*) and (**b**) HMG-domain (encoded by *MAT1-2-1*). Bootstrap support values (≥ 48%) are indicated at each node. The trees were rooted using sequences from *N. castellii* (Saccharomycotina) and *R. stolonifer* (Mucoromycota) as outgroups. Multiple sequence alignments performed by MUSCLE of mating-type proteins conserved regions: (**c**) α-domain and (**d**) HMG-domain. Sequences correspond to both representative taxa of truffle-forming Pezizomycetes and closely related taxa. Residues are colored by conservation score, with dark blue indicating high conservation (BLOSUM 62 matrix). Conservation scores are plotted below each alignment. Red bars mark putative intron positions, inferred from alignment gaps and domain structure annotations

InterPro analysis revealed the presence of two conserved domains within the amino acid sequences of the mating type proteins: an α-box domain with 58 aas in TcMAT1-1-1 and an HMG-box domain with 78 aas in TcMAT1-2-1 (Supplementary Fig. 6). These sequences were used to investigate their evolutionary relationships among various species within the Pezizomycetes class (Fig. 3). The α-box domain neighbour-joining tree showed that the studied genera belonging to Pezizaceae family considered as desert truffles (*Terfezia*, *Tirmania*, *Mattirolomyces*, and *Kalaharituber*), formed a single, well-supported clade (99). In the HMG-box domain tree, *Terfezia* and *Kalaharituber* clustered together with a bootstrap value of 77. The genus *Picoa*, which belongs to the Pyronemataceae family but is also regarded as a desert truffle, was positioned outside this clade. In both trees, as expected, the different *Tuber* species (Tuberaceae) appeared closely related, forming well-supported clusters. Interestingly, the α- and HMG-box domain trees show clustering patterns similar to those obtained with the ITS-based phylogenetic tree of the studied species (Supplementary Fig. 7).

The conserved regions of TcMAT proteins were aligned with those from various Pezizomycetes species (Fig. 3C-D). In total, 10 residues in α-box domain and 17 in HMG domain alignments were fully conserved across all analysed species. Additionally, a conserved intron position was observed across all species, corresponding to a codon encoding serine or cysteine residue within the α-box domain alignment (Fig. 3C). However, for the HMG-domain, the number and position of introns varied between species, ranging from two to four (Fig. 3D). This variability indicates greater structural divergence according to the intron gain or loss events in the evolution of the *MAT1-2-1* gene compared to *MAT1-1-1* gene.

**Fig. 4:**
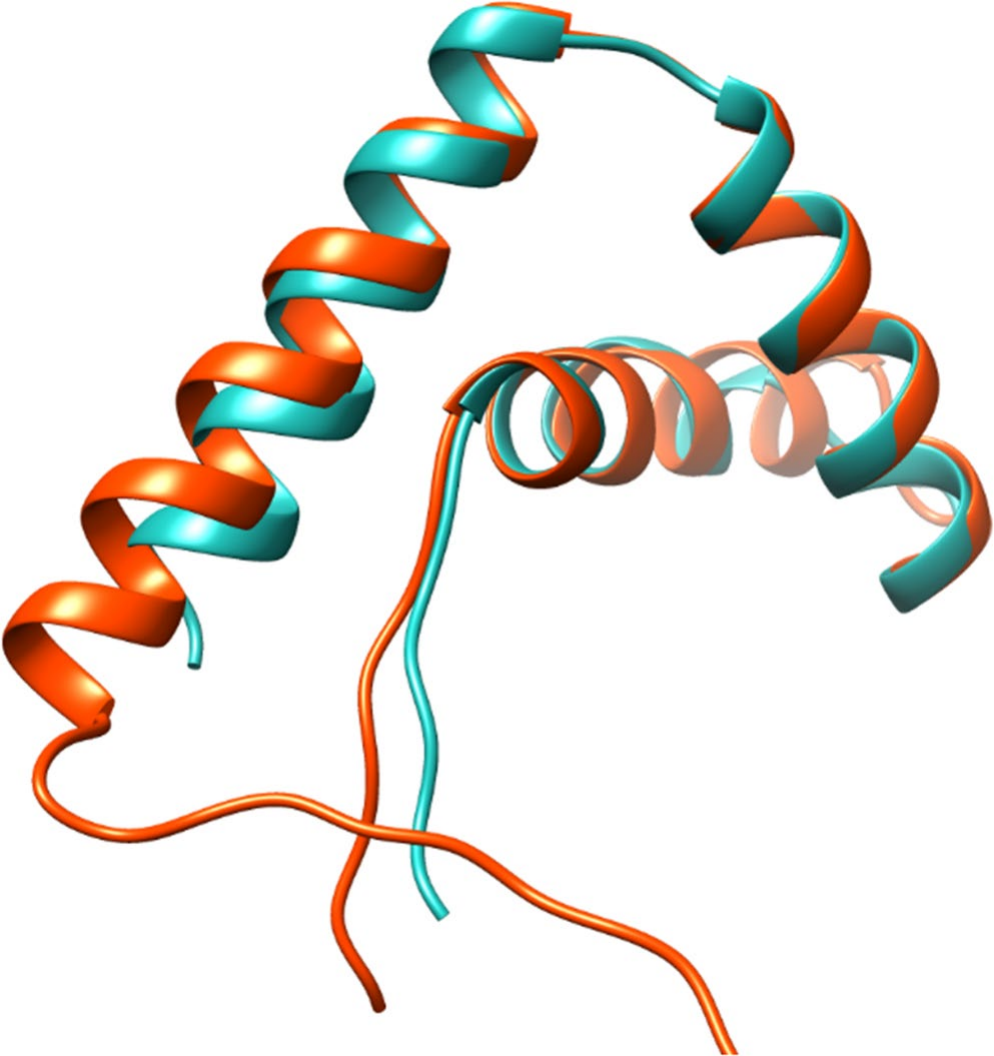
Superposition of the tertiary structure of the conserved α- (blue) and HMG-box (red) domains of TcMAT1-1-1 and TcMAT1-2-1 proteins

The sequences of proteins encoded by *TcMAT1-1-1* and *TcMAT1-2-1* allowed the study of the tertiary structures of the α- and HMG-box domains, which were predicted using AlphaFold Server. For the α-box domain, the confidence score was predicted template modelling pTM = 0.72, while for the HMG-box domain, the predicted confidence was pTM = 0.78, indicating moderate to high structural reliability in both cases (Xu and Zhang 2010). According to MolProbity, the best model for each domain was selected and subsequently superimposed for structural comparison (Fig. 4). Their three-dimensional structures exhibited remarkable similarity, as evidenced by an RMSD value of 0.78 Ångströms when aligned. This low RMSD value indicates excellent structural conservation between the α- and HMG-box domains, suggesting they adopt nearly identical protein folds despite their sequence divergence. The structural similarity observed supports both a functional relationship and a potential evolutionary connection between these mating-type domains and their potential role in transcriptional regulation within the mating system of this organism.

Although amino acid conservation across the aligned sequences was variable (Fig. 3C-D), evolutionary analysis of mating type genes across Pezizomycetes species using the FEL method revealed a predominant pattern of purifying selection. For *MAT1-1-1*, 55 out of 158 variable codons (≈ 34.8%) exhibited significant evidence of purifying selection (dN/dS < 1; *p* < 0.05). Similarly, for *MAT1-2-1*, 46 out of 119 codons (≈ 38.7%) were under purifying selection at the same significance threshold. Importantly, no codons were identified under diversifying (positive) selection, indicating strong evolutionary constraint on both genes.

### Spatio-temporal evolution of *TcMAT* genes

**Fig. 5:**
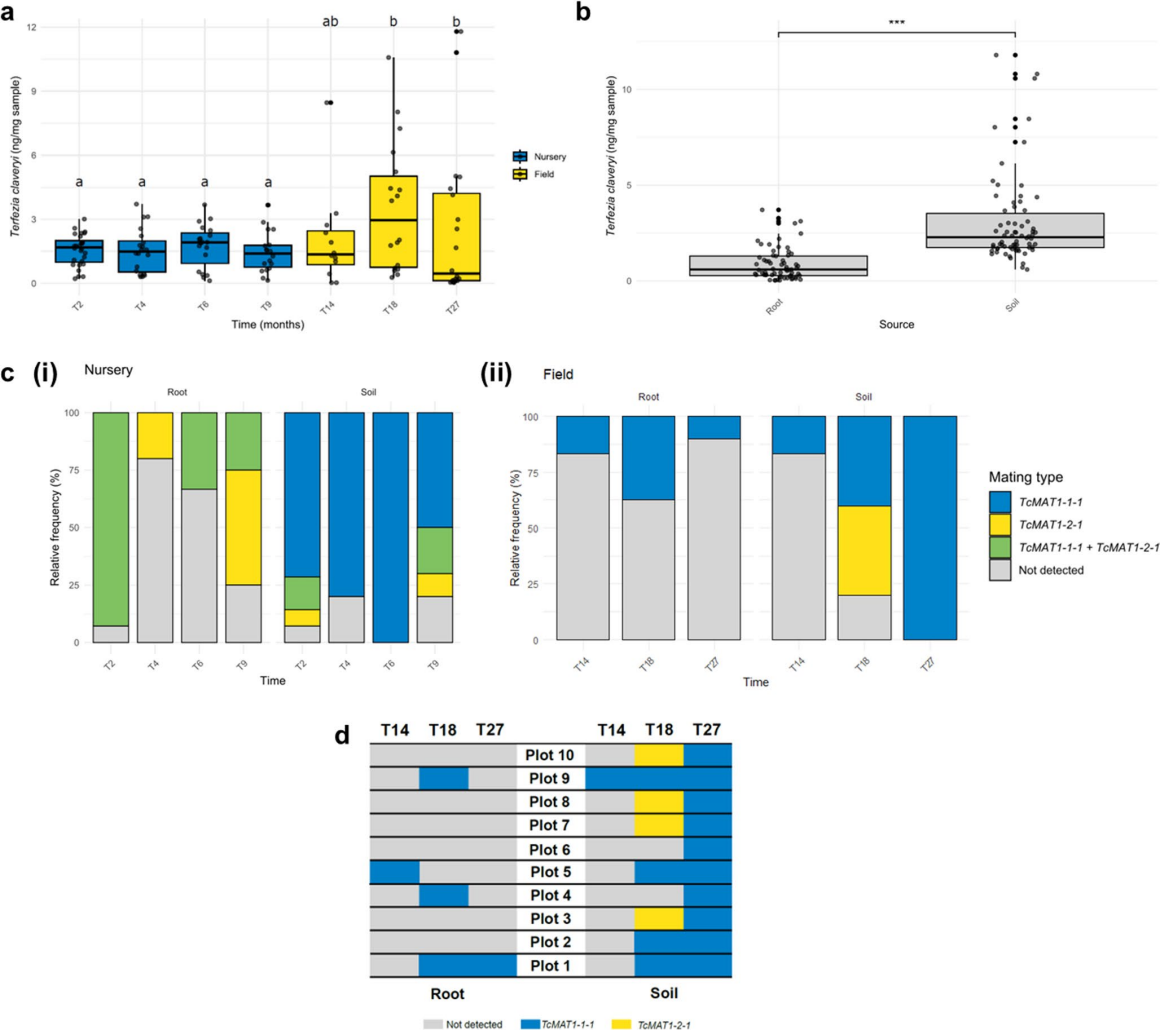
Evaluation of the distribution of *T. claveryi* and its mating idiomorphs. (**a**) Amount of *T. claveryi*, expressed as the square root of ng/mg of sample, along the 7 time points of study in all root and soil samples; (**b**) amount detected of *T. claveryi*, represented as the square root of ng/mg of sample, according to the source (root or soil) for all the different samples collected over the study period; (**c**) relative frequency of *TcMAT* genes in root and soil samples across the time points according to their location: (i) nursery and (ii) field; (**d**) *TcMAT* distribution across the different plots of the sampling plantation. T2-T9 and T14-T27 correspond to sampling times in the nursery and in the field stages, respectively

Over a two-year period, the dynamic of *T. claveryi* from nursery to the field was followed. *T. claveryi* was detected and quantified by qPCR in all root and soil samples, confirming the persistence of the fungus across the complete sampling timeline (Fig. 5A). In the nursery scenario, the abundance of *T. claveryi* remained relatively stable during the nine months following inoculation. This pattern was maintained to time T14, which corresponds to the first samples collected from field two weeks after transplantation. Interestingly, a marked increase in variability was observed at subsequent times T18 and T27, as evidenced by a broader distribution in fungal abundance values. Statistical analysis revealed significant differences in *T. claveryi* abundance between time T18 and all preceding nursery time points (*p-values*: T2–T18 = 0.001, T4–T18 = 0.0055, T6–T18 = 0.0047, T9–T18 = 0.0006), and also between time T27 and all nursery time points (*p-values*: T2-T27 = 0.0093, T4-T27 = 0.0411, T6-T27 = 0.026, T9-T27 = 0.0133). Additionally, *T. claveryi* abundance differed significantly according to the sample source (soil - roots; *p-value* = 0.0004; (Fig. 5B). Specifically, fungal quantity was consistently lower in root than in soil samples.

When the frequence of *TcMAT* genes was investigated, differences according to the source and location variables emerged (Fig. 5C). Whereas in root samples from nursery plants both *TcMAT1-1-1* and *TcMAT1-2-1* were detected, root samples from the field plants exhibited the *TcMAT1-1-1* only, regardless of the collection timing. *TcMAT1-1-1*, *TcMAT1-2-1* and their combination (*TcMAT1-1-1* + *TcMAT1-2-1*) were detected in soil nursery samples, with *TcMAT1-1-1* showing the highest frequency. In soil field samples both *TcMAT1-1-1* and *TcMAT1-2-1* were detected, but not their combination. In addition, the abundance of *TcMAT* genes in field was evaluated (Fig. 5D). *TcMAT1-1-1* was found in all the 10 plots analysed, whereas *TcMAT1-2-1* was present only in the 40% of the plots.

## Discussion

By combining in silico and molecular approaches, here we not only provide compelling evidence in support of the heterothallic reproductive mode of *T. claveryi*, but also shed preliminary lights on the spatio-temporal distribution of *T. claveryi* strains of opposite mating type on both host plants and soil. This information along with the protocols herein developed are crucial to drive management practices to promote desert truffle spreading and fructification. Additionally, first hints on the evolution within Pezizomycete class of *MAT* genes and regulatory motifs controlling their expression have been provided.

### Detection of *TcMAT* genes requires a nested PCR approach

Attempts to detect the single-copy *TcMAT* genes using conventional PCR proved challenging since the amount of *T. claveryi* in soil and root samples was considerably low, especially in roots. Other Pezizomycetes fungi, such as the ectomycorrhizal *Tuber magnatum*, exhibit low levels of root colonization combined with more extensive extraradical mycelium in soil (Murat et al. 2005). Our results support that *T. claveryi* may follow the same pattern, being more abundant in the soil compared to roots. Moreover, field observations and previous studies in *T. claveryi* suggest that its low abundance in the root environment despite its initial inoculation with the host plant may also be explained by competitive interactions with other soil fungi, which could outcompete this species over time, as previously reported by Arenas et al. (2021). Consequently, a nested PCR protocol was developed and optimized to enhance sensitivity and minimize the occurrence of false positives. Nested PCR, which involves two sequential rounds of amplification using two sets of primers, was found to be highly sensitive and reliable specially in soil samples. The greatest difficulties in amplifying these genes were encountered in field root samples, which is consistent with the low concentration of *T. claveryi* DNA found in this tissue. Using nested PCR on roots to detect *T. claveryi* DNA concurs with previous reports in other species sharing ecological niche, such as *Picoa* spp. (Jamali and Banihashemi 2013) and provides valuable insights into the biology of these species.

### Sexual reproduction in *T. claveryi* requires outcrossing

As already suggested by Marqués-Gálvez et al. (2021), our work confirms that the *MAT* locus in *T. claveryi* is constituted by two non-concurrent idiomorphs, *TcMAT1-1* and *TcMAT1-2*, each one characteristic of specific strains. This research represents the first report of a desert truffle species for which the mating-type reproductive mode has been characterised.

The detection and sequencing of different mating type idiomorphs in individual spores derived from the same ascus supports the haploid nature of *T. claveryi* ascospores. This observation indicates successful segregation of the *MAT* locus during meiosis, which is typical of heterothallic ascomycetes (Wilson et al. 2021). The confirmation of haploidy at the spore level reinforces the hypothesis that mating compatibility in *T. claveryi* requires the interaction of two genetically distinct strains carrying opposite mating types. This finding is consistent with previous reports in the *Neurospora crassa* model ascomycetes (Raju 1992) as well as other mycorrhizal truffle species such as *Tuber melanosporum* (Rubini et al. 2011a), *Tuber indicum* (Belfiori et al. 2013) or *Tuber borchii* (Belfiori et al. 2016). Moreover, it suggests that under natural conditions, ascospore germination and subsequent mycelial development are likely initiated from a single mating type, thus requiring the presence of a compatible strain in the surrounding environment to complete the sexual cycle. In this scenario, periodic selection and inoculation of host plants with sexually compatible strains of *T. claveryi*, specially in field stage, may notably increase the likelihood of mating and, therefore, fruiting body development (Rubini et al. 2011a, 2014; Molinier et al. 2016; De la Varga et al. 2017).

### The genetic structure of *T. claveryiMAT* locus reveals lineage-specific evolution and specific regulatory mechanisms

The characterisation of *MAT* locus through RNAseq and DNA sequencing enabled the elucidation of its genomic structure. Interestingly, *TcMAT1-1* and *TcMAT1-2* idiomorphs are 2,868 bp and 3,087 bp in length, respectively. This contrasts with other hypogeous symbiotic ascomycetes, such as *T. melanosporum*, in which both *MAT1-1* and *MAT1-2* idiomorphs are considerably longer, consisting of 7,430 bp and 5,550 bp, respectively (Rubini et al. 2011a). The *MAT* idiomorphs lengths in other *Tuber* species are even greater (Belfiori et al. 2016). Differently from the studied *Tuber* species (Rubini et al. 2011a; Belfiori et al. 2016), in the two *T. claveryi MAT* idiomorphs neither transposon-like elements nor additional ORFs are present. The strands of *TcMAT* genes also reveals a different structural organization when compared to other described Pezizomycetes. Both *TcMAT1-1-1* and *TcMAT1-2-1* are in the same strand. This configuration contrasts with that of other truffles species such as *T. indicum* and *T. melanosporum*, where *MAT1-1-1* shares the same strand but *MAT1-2-1* is located on the opposite strand (Rubini et al. 2011a; Belfiori et al. 2013). Conversely, in *T. borchii*, *MAT1-1-1* displays different strand relative to *T. claveryi* (Belfiori et al. 2016).

The multiple sequence alignment of the conserved α- and HMG-box domains across various Pezizomycetes species revealed a high degree of conservation, with a notable number of aligned residues belonging to chemically similar amino acid groups. This outcome implies that most of the observed substitutions are conservative, preserving the structural or functional integrity of these domains and indicating potential importance across different species. Remarkably, a conserved intron at DNA level is present in the same position in all organisms within the α-box domain, although the corresponding amino acid alternates between cysteine (*T. claveryi*, *Tirmania nivea*, *Mattirolomyces terfezioides*) and serine (*Kalaharituber pfeilli*, *Ascobolus immersus*, *T. melanosporum*, *T. borchii*, *T. indicum*). These residues are structurally similar in size and polarity but differ in chemical properties, since cysteine contains a thiol group, which is highly reactive and can form disulfide bonds, whereas serine has a hydroxyl group, which is less reactive. In contrast, the HMG-box domain displays variation in both the number and location of introns among species. Altogether, the mentioned variations may reflect lineage-specific adaptations (Yampolsky and Stoltzfus 2005; Huzurbazar et al. 2010). Such molecular phenomenon plays a role in the evolutionary dynamics of mating-type loci, possibly contributing to the suppression of recombination and the regulation of gene expression (Idnurm et al. 2008; De Hoff et al. 2013; Yamazaki et al. 2017).

Regarding gene regulation, several DNA-binding motifs were identified in the promoter regions of both genes. Importantly, IME1, which is present in several copies, and other motifs (e.g. RME1, ASH1, MCM1, SWI4, NDT80 or SUM1) are associated with transcription factors involved in biological functions such as sexual reproduction, cell cycle progression, response to stimuli and development. This is consistent with the known function of the *MAT* locus in fungi, which orchestrates the regulation of reproductive processes (Fraser and Heitman 2003; Debuchy et al. 2010). Additionally, other binding motifs related to stress response (STB3, MAC1, IXR1, HSF1, ARR1) and aminoacidic metabolism (CHA4, LEU3) were also identified. The presence of these motifs suggests that sexual reproduction in *T. claveryi* may be activated under stressful or changing environmental conditions, consistent with the idea that fungi tend to shift to sexual reproduction as an adaptive response to stress (Schoustra et al. 2010). Amino acid availability could play a critical role in regulating this process in *T.claveryi* as much as in *Aspergillus nidulans*, whose sexual development is promoted when amino acids are abundant and repressed under amino acid starvation (Hoffmann et al. 2000). Therefore, while environmental stress may act as a signal to initiate sexual reproduction, sufficient nutrient availability, particularly of amino acids, might be required to complete the process successfully. This possible coordinated regulation highlights the adaptive flexibility of *T. claveryi*, allowing it to integrate environmental and metabolic signals to optimize sexual reproduction and fruiting body formation under arid conditions. This finding is essential for better understanding desert truffle production. Interestingly, the YPR196W motif, bound by transcription factors which may be involved in maltase and maltose permease transcription genes is present in both *MAT1-1-1* and *MAT1-2-1* promoters, suggesting a potentially conserved regulatory role.

The presence of an intron in the 5’UTR of *TcMAT1-2-1* suggests that this gene may possess specialized regulatory features (Bicknell et al. 2012; Hoshida et al. 2017). This intron can harbour transcription factor binding sites and influence gene expression at multiple levels, from transcription to translation. Evidence from *Arabidopsis* indicates that 5’UTR introns can enhance mRNA accumulation and modulate transcription start site selection, supporting their role as regulatory elements (Chung et al. 2006; Gallegos and Rose 2017). In *TcMAT1-2-1*, this intron may provide an additional layer of control, ensuring precise temporal and spatial expression, which could be crucial for sexual differentiation and adaptive responses to environmental cues in *T. claveryi*. In contrast, *TcMAT1-1-1* only contains an intron, which is located in the coding region, signifying a simpler mechanism. The structural difference may reflect distinct regulatory requirements between the two mating types. Further studies should be aimed to experimentally validate the TF-DNA interactions here described in the promoter regions of *TcMAT* genes, which will help to better understand the regulatory mechanisms that trigger sexual reproduction in truffles.

Previous comparative analyses suggest that in Pezizomycotina, the α-box domain present in MAT1-1-1 evolved from an ancestral MATA_HMG domain within the HMG-box superfamily and retained its tertiary conformation due to functional constraints (Martin et al. 2010). Our results in *T. claveryi* are in line with this report. We showed that the amino acid sequences of the α- and HMG-box domains in *T. claveryi* have diverged considerably (similarity = 34.1%). However, their predicted tertiary structures are highly similar, as evidenced by a low RMSD value. This finding implies that both domains share a conserved fold which is characteristic of the HMG-box superfamily, since they have similar L-shaped architecture with three α-helices. The preservation of the same scaffold in both domains suggests that they perform similar biological roles, from transcriptional regulation to mating-type determination (Ait Benkhali et al. 2013). Maintenance of the tertiary structure despite notable differences in primary sequence is a well-known phenomenon, particularly in DNA binding proteins and protein-protein interactions (Illergård et al. 2009; Thapar 2015). Therefore, the structural conservation despite the low sequence similarity within α- and HMG-box domains observed in TcMAT proteins suggests that selective pressure act primarily on preserving structural integrity, rather than on their exact amino acid composition. Importantly, selection analysis revealed significant finding of purifying selection, indicating that there is a strong selective pressure to eliminate non-synonymous mutations that alter the conserved domains function not only for *T. claveryi*, but also for the analysed Pezizomycetes. This extreme conservation of tertiary structure is likely due to the critical role of MAT proteins as master regulators in reproductive mechanisms, where structural changes could compromise recognition specificity between strains (Fraser and Heitman 2003; Debuchy et al. 2010; Ait Benkhali et al. 2013). Overall, our findings provide functional insight into the molecular mechanisms of fungal mating-type regulation and adds strong evidence related to the hypothesis of a common ancestral MATA_HMG domain as an earlier form of fungal *MAT* loci, which gave origin to the current α- and HMG-box domains, first postulated by Idnurm et al. (2008) and supported by Martin et al. (2010).

### Mating type fingerprinting of *H. almeriense* x *T. claveryi* reveals tissue-related limitations and a loss of mating diversity from nursery to field

The deep characterization of both *TcMAT* genes allowed the design of specific primers and the optimization of a protocol to detect the sexual identity of *T. claveryi* in different organs. As a case scenario, we evaluated the presence of the idiomorphs in a set of mycorrhizal roots of *H. almeriense* and the surrounding soil.

Regarding *T. claveryi* concentration, the relatively low and uniform presence of the fungus during the nursery phase suggests that the controlled conditions constrained fungal growth, maintaining a balanced symbiotic relationship (Talley et al. 2002; Jacquemyn et al. 2015). However, this pattern changed after the transplantation of plants to the field, where a marked increase in fungal DNA concentrations was observed at T18 and T27, albeit with a large variability among samples. Such phenomenon observed in field may be influenced by microenvironment heterogeneity and the adaptation of the symbiosis to the new conditions (Bang et al. 2018; Arenas et al. 2022b). Altogether, these outcomes highlight how environmental factors can promote or disturb fungal colonization dynamics beyond controlled nursery settings.

Determination of *TcMAT* genes in nursery and field root samples was limited due to the low abundance of *T. claveryi* DNA, as previously mentioned, likely leading to underestimation of idiomorph presence in organ. Although a mix of spores from different mating type were introduced into the substrate during inoculation, root colonization and mycorrhiza formation require time, and fungal biomass within the roots remained low during the early months, making its detection complicated. Even in the field stage, detection in roots can be constrained by climatic conditions or direct competition with other fungi, which may limit the establishment and proliferation of *T. claveryi* (Arenas et al. 2021). Since the sensitivity of the primers employed in the nested PCR is comparable for both *TcMAT* genes (Supplementary Fig. 8), the aforementioned factors, together with the single-copy nature of *TcMAT* genes, likely contributed to the reduced detection sensitivity.

According to the mating type distribution at nursery stage, both *TcMAT1-1* and *TcMAT1-2* idiomorphs were found in root samples, with frequencies fluctuating over time. In contrast, the soil compartment exhibited a predominance of *TcMAT1-1-1*, which was consistently detected across all sampling points, indicating its early establishment or greater sensitivity advantage. In the field stage, the pattern became even more pronounced, as most of the determined samples were dominated by *TcMAT1-1-1*. The frequency of *TcMAT1-2-1* was substantially reduced, and in some time points, not detected. Importantly, the use of spores as inoculum ensures that both mating types are provided to the host plants. In line with this, strains of both mating types are found under controlled conditions, as shown by root samples collected at T2-T9. In contrast, after transplantation, a specific mating type (*MAT1-1-1*) became dominant on the roots of host plants. Interestingly, this mating type is the same that prevailed in soil nursery samples. This striking shift towards a single mating type suggests that the balance between idiomorphs depends on many, and still unknown variables governing the different steps of plant and soil colonization by *T. claveryi*. In turn, this observation let us to argue that strains harboring opposite *MAT* idiomorphs respond differently to identical environmental conditions, likely due to different regulation of *MAT* genes or *MAT-*responsive loci. Polymorphism in the promoter regions of the *MAT* genes and/or the presence of a 5’UTR intron in *TcMAT1-2-1* may be responsible for the more restricted spatio-temporal distribution of strains carrying the *MAT1-2-1* with respect to those carrying the *MAT1-1-1* gene. Therefore, an intron-mediated regulation, polymorphisms within the regulatory regions of *MAT* genes or a combination of both factors may influence the distinct distribution patterns of strains of opposite mating types. Further studies are warranted to elucidate the relative contribution of these mechanisms.

The bias distribution of *T. claveryi* mating types may have significant implications for the sexual reproduction potential and the maintenance of genetic diversity in *T. claveryi* populations under field conditions. In fact, no ascocarp production was observed during the field stage, supporting the hypothesis that biased mating type distributions limit reproductive success. Considering that desert truffle production usually begins three years after planting, this timeframe may correspond to the period required for compatible strains carrying the alternate *MAT* to colonize neighboring plants or soil and enable ascocarp formation.

Altogether, it appears that colonization in the studied field is highly selective or that *TcMAT1-1-1* is more competitive during the colonization process. This finding goes hand with hand with similar results reported in *T. melanosporum* open-field studies (Rubini et al. 2011b), where a notable prevalence of a specific mating type was observed at individual sites. Thus, the dominance of one mating type over the other may depend on (i) the competitiveness of each strain regardless its *TcMAT* gene or (ii) the sensibility of each gene based on polymorphisms. Indeed, the distribution of mating type genes is often non-random and influenced by both ecological and cultivation factors (Murat et al. 2013; De la Varga et al. 2017). Further research in several plantations is required to elucidate specific patterns that determine the selectivity of an idiomorph over the other.

Natural truffle populations, including desert truffles, tend to be spatially structured, with patches dominated by a single mating type which can limit the potential for sexual reproduction. In this context, the potential use of desert truffle nests to balance mating type distribution, as it has been already tested in black truffes (Garcia-Barreda et al. 2020), remains largely unexplored and could represent a promising strategy to enhance cultivation yield. These patterns presented here give insight about the distribution and persistence of mating types in ectendomycorrhizal fungi, and suggest further research is necessary to clarify the mechanisms driving idiomorph dominance and its fructification consequences. In this context, it would be interesting study more *H. almeriense* x *T. claveryi* plantations in more diverse contexts and during longer periods of time.

Monitoring *TcMAT* genes in both roots and soil provides valuable information for optimising desert truffle cultivation. The identification of the *MAT* idiomorph that predominates in a plantation could allow to introduce additional inoculum containing strains of the opposite mating type in order to restore balance and increase the probability of sexual reproduction and fruiting body formation. Therefore, the methodology established in this work represents a key tool for improving the management and productivity of this type of cultivation systems.

## Conclusion

The present study provides meaningful insights into the reproductive system of *T. claveryi* and sheds light on the phylogenetic relationships and evolution of *MAT* genes in symbiotic Pezizomycetes. The finding that the two master genes for sexual reproduction (*MAT1-1-1* and *MAT1-2-1*) are present in different strains provides conclusive evidence that *T. claveryi* is a heterothallic fungus. This observation, coupled to the uneven distribution in the field of strains carrying opposite mating types, should lead to a serious reconsideration of the process of inoculating of host plants and orchard management practices, with the aim of promoting desert truffle fruiting.

## Supplementary Information

Below is the link to the electronic supplementary material.

Supplementary Material 1

Supplementary Material 2

Supplementary Material 3

Supplementary Material 4

## Data Availability

All data supporting the findings of this study are available within the paper and its Supplementary Information.
