# Supplementary material for: Terfezia claveryi MAT locus characterization uncovers evolutionary insights about sexual reproduction of Pezizomycetes and reveals mating type dynamics in mycorrhizal plants
Source: Mycorrhiza. 2026 May 7;36(3):21. doi: 10.1007/s00572-026-01266-3 (PMC13152902; doi:10.1007/s00572-026-01266-3)
Supplement: Supplementary file 1 — Supplementary Material 1 [file 572_2026_1266_MOESM1_ESM.docx]

**Supplementary methods**

***Terfezia claveryi* *MAT* locus characterization uncovers evolutionary insights about sexual reproduction of Pezizomycetes and reveals mating type dynamics in mycorrhizal plants.**

Laura Andreu-Ardil^1^, Ángel Guarnizo^1^, Alfonso Navarro-Ródenas^1^, Francisco Arenas^1^, Manuela Pérez-Gilabert^2^, José Eduardo Marqués-Gálvez^1^*, Francesco Paolocci^3†^, Asunción Morte^1^*^†^

^1^Departamento de Biología Vegetal (Botánica), Facultad de Biología, Universidad de Murcia, Campus de Espinardo, Murcia 30100, Spain

^2^Departamento de Bioquímica y Biología Molecular-A, Universidad de Murcia, Campus de Espinardo, Murcia 30100, Spain

^3^CNR-IBBR, Istituto di Bioscienze e Biorisorse, UOS di Perugia, Perugia 06128, Italy

* Corresponding authors: José Eduardo Marqués-Gálvez ([joseeduardo.marques@um.es](mailto:joseeduardo.marques@um.es)), Asunción Morte ([amorte@um.es](mailto:amorte@um.es))

^†^These authors contributed equally as last senior authors.

**Supplementary methods 1. Optimization of the PCR protocol for DNA samples.**

Several attempts were made to amplify *TcMAT* genes from DNA samples using Phusion™ High - Fidelity DNA Polymerase (Thermo Fisher Scientific Baltics UAB, Lithuania).

Two main PCR protocols were used. On the one hand, the two-step protocol consisted of an initial denaturation at 98 °C for 30 seconds, 35 cycles of (i) denaturation at 98 °C for 7 seconds and (ii) combined annealing/extension step at 72 °C for 2 minutes, and a final extension at 72 °C for 7 minutes. On the other hand, the three-step protocol included an initial denaturation at 98 °C for 30 seconds, followed by 35 cycles of (i) denaturation at 98 °C for 7 seconds, (ii) annealing at 68 °C for 10 seconds, and (iii) extension at 72 °C for 30 seconds, and an extension at 72 °C for 7 minutes.

For the detection of sexual genes in DNA extracted from *in vitro* cultured free-living mycelium, the three-step PCR was conducted in a 20 μl reaction containing 0.2 mM of each dNTP, 0.2 μM of each primer (MAT111Fwd-MAT111Rev, MAT111FWDALT- MAT111Rev, MAT111BFwd- MAT111BRev and MAT121Fwd- MAT121Rev), and 10 ng of DNA. Reactions were not multiplexed. Then, different annealing temperatures and concentrations of the reagents were tested. However, no amplification was observed under these conditions. As consequence, a nested PCR was performed.

The first reaction followed the two-step procedure using the already mentioned reagent concentrations and 50 ng of template harbouring *TcMAT1-1-1* or *TcMAT1-2-1*. Primers 453-455 were used in this round of amplification. Then, for *TcMAT1-1-1* amplification, the MAT111Fwd-MAT111Rev, MAT111FWDALT-MAT111Rev, and MAT111BFwd-MAT111Brev primer pairs were evaluated in a nested PCR targeting 10 ng of the PCR products following the two-step protocol. Double bands in *TcMAT1-1-1* positive samples were detected in reactions using MAT111Fwd-MAT111Rev and MAT111FWDALT- MAT111Rev, leading to the exclusion of these combinations from further analysis. A single band of the expected size in samples containing *TcMAT1-1-1* was observed with MAT111BFwd-MAT111BRev. Nevertheless, this primer pair also produced slight amplification for samples harbouring *TcMAT1-2-1*. To address this issue, a three-step PCR was performed for the second reaction and PCR products from the first reaction were purified using the GeneJET PCR Purification Kit (Thermo Fisher Scientific Baltics UAB, Lithuania), following the manufacturer's instructions. Initially, the annealing temperature was set to 64 °C. However, increasing it to 66 °C improved amplification. After these modifications, no false positives were detected. These optimized conditions were also implemented for the amplification of *TcMAT1-2-1*, where a three-step nested PCR using MAT121Fwd-MAT121Rev primer pair was performed using 10 ng of the purified PCR products from the first PCR. No amplification for *TcMAT1-1-1* was observed in this case, but a clear band of the expected size was detected in *TcMAT1-2-1* positive samples.

Taking these assays into account, a multiplex PCR was performed in the second reaction. Results did not differ from those obtained with conventional PCR.

This PCR protocol was also valid for environmental DNA samples. However, the input DNA concentration for the first PCR was optimized. Reactions with DNA concentrations ranging from 50 ng to 150 ng were tested. Subsequently, the resulting PCR products were purified and used in a multiplex nested PCR with MAT111BFwd-MAT111BRev and MAT121Fwd-MAT121Rev, where template concentrations ranged from 2.5 ng to 10 ng. Optimal results were obtained using 50 ng of DNA in the first PCR and 2.5 ng in the nested reaction.

Multiplex PCR reaction was not consistent with spores DNA samples. Thus, MAT genes in these samples were analysed using a standard nested PCR under the previously described conditions.
