# Supplementary material for: Terfezia claveryi MAT locus characterization uncovers evolutionary insights about sexual reproduction of Pezizomycetes and reveals mating type dynamics in mycorrhizal plants
Source: Mycorrhiza. 2026 May 7;36(3):21. doi: 10.1007/s00572-026-01266-3 (PMC13152902; doi:10.1007/s00572-026-01266-3)

Suplementary Dataset 1. Uncropped gels and blots image(s) related to Figure 1 and Supplementary Figure 8

Terfezia claveryi MAT locus characterization uncovers evolutionary insights about sexual reproduction of Pezizomycetes and reveals mating type dynamics in mycorrhizal plants.

Laura Andreu-Ardil[1], Ángel Guarnizo[1], Alfonso Navarro-Ródenas[1], Francisco Arenas[1], Manuela Pérez-Gilabert[2], José Eduardo Marqués-Gálvez[1]*, Francesco Paolocci[3†], Asunción Morte[1]*[†]

[1]Departamento de Biología Vegetal (Botánica), Facultad de Biología, Universidad de Murcia, Campus de Espinardo, Murcia 30100, Spain

[2]Departamento de Bioquímica y Biología Molecular-A, Universidad de Murcia, Campus de Espinardo, Murcia 30100, Spain

[3]CNR-IBBR, Istituto di Bioscienze e Biorisorse, UOS di Perugia, Perugia 06128, Italy

* Corresponding authors: José Eduardo Marqués-Gálvez (joseeduardo.marques@um.es), Asunción Morte (amorte@um.es)

[†]These authors contributed equally as senior authors.

Uncropped gel from Figure 1a

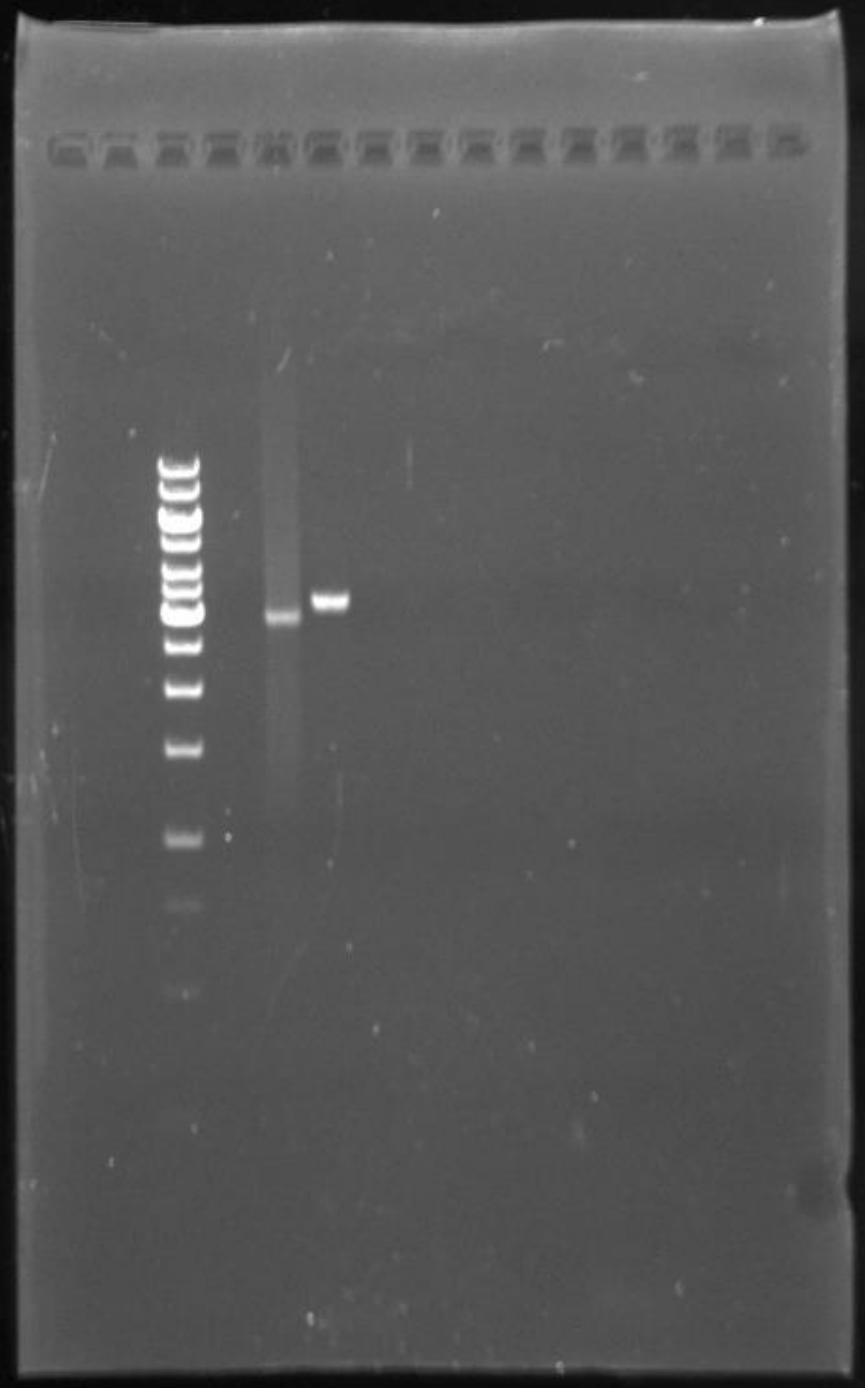

Uncropped gel from Figure 1b and 1c

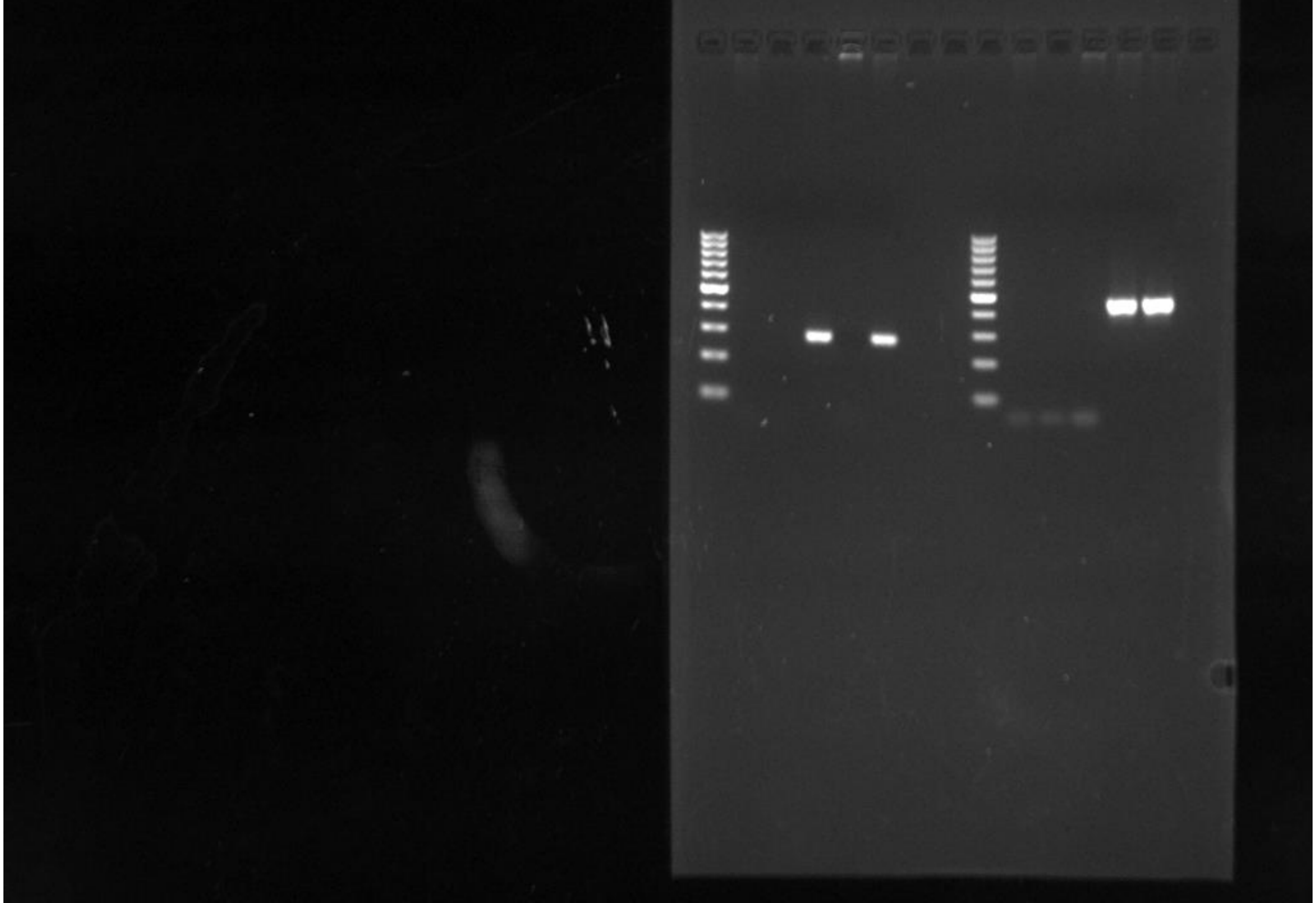

Uncropped gel from Figure 1d

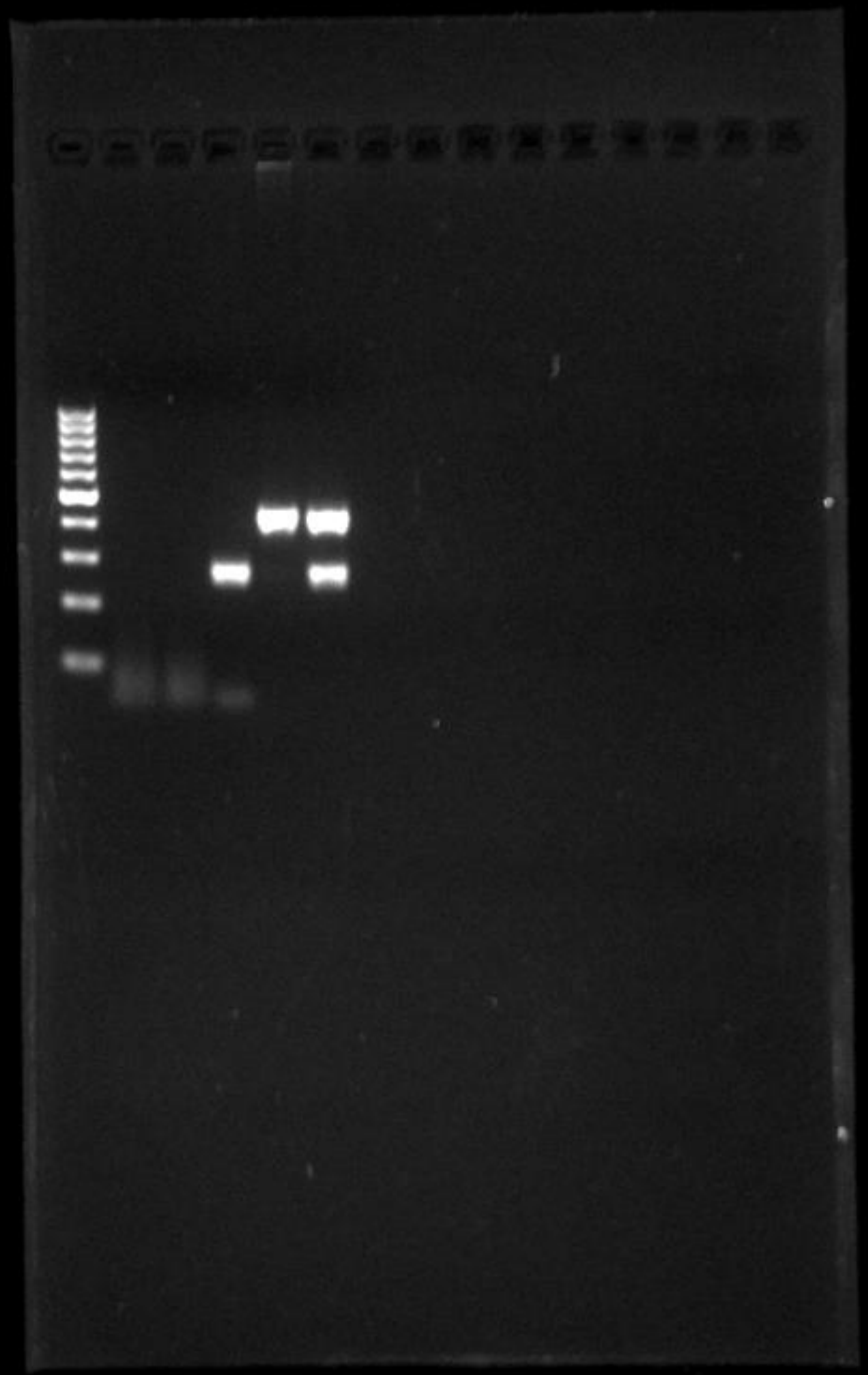

Uncropped gel from Figure 1e

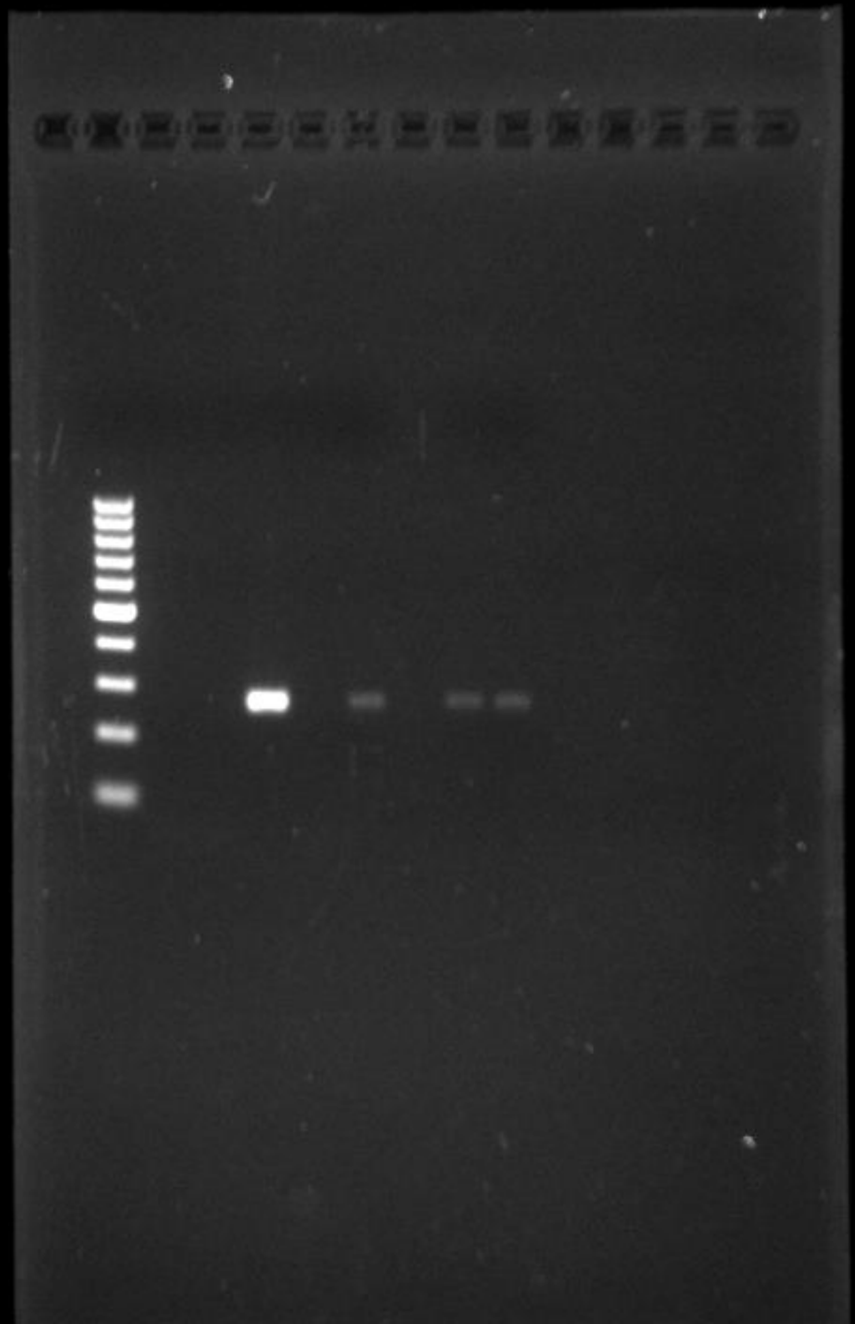

Uncropped gel from Figure 1f

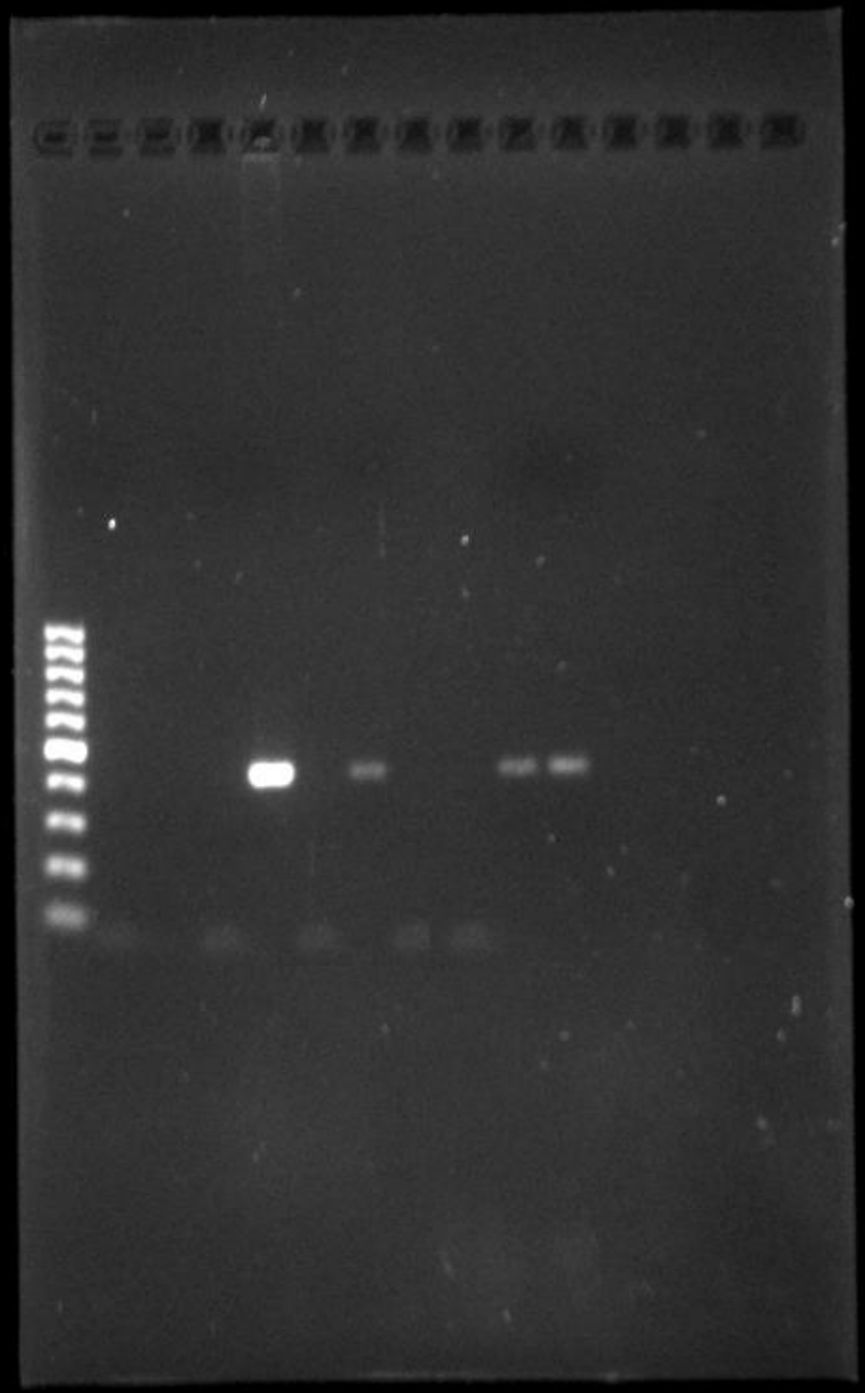

Uncropped gel from Supplementary Figure 8

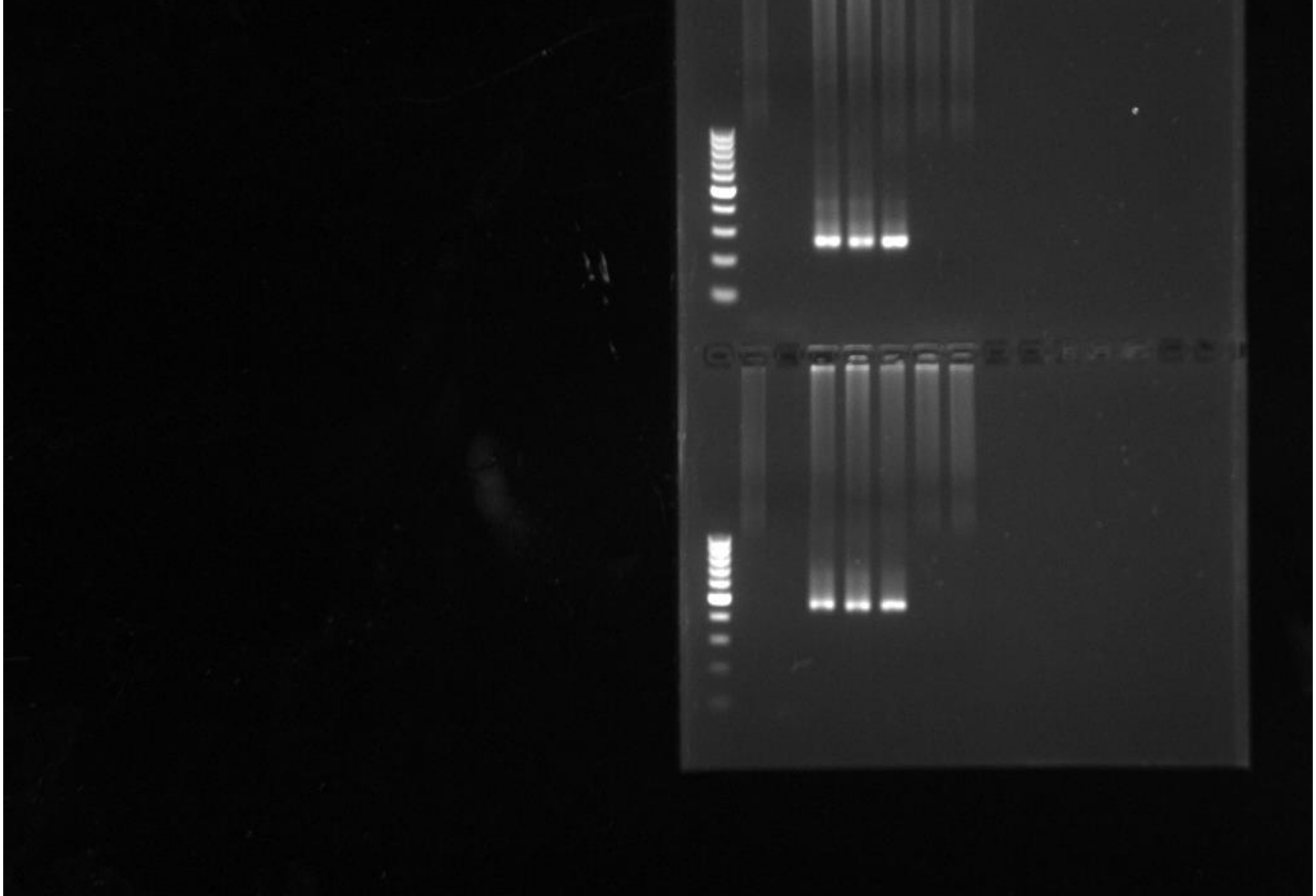

Supplement: Supplementary file 3 — Supplementary Material 3 [file 572_2026_1266_MOESM3_ESM.pdf]
