# Supplementary material for: Terfezia claveryi MAT locus characterization uncovers evolutionary insights about sexual reproduction of Pezizomycetes and reveals mating type dynamics in mycorrhizal plants
Source: Mycorrhiza. 2026 May 7;36(3):21. doi: 10.1007/s00572-026-01266-3 (PMC13152902; doi:10.1007/s00572-026-01266-3)
Supplement: Supplementary file 4 — Supplementary Material 4 [file 572_2026_1266_MOESM4_ESM.docx]

**Suplementary Figures**

***Terfezia claveryi* *MAT* locus characterization uncovers evolutionary insights about sexual reproduction of Pezizomycetes and reveals mating type dynamics in mycorrhizal plants.**

Laura Andreu-Ardil^1^, Ángel Guarnizo^1^, Alfonso Navarro-Ródenas^1^, Francisco Arenas^1^, Manuela Pérez-Gilabert^2^, José Eduardo Marqués-Gálvez^1^*, Francesco Paolocci^3†^, Asunción Morte^1^*^†^

^1^Departamento de Biología Vegetal (Botánica), Facultad de Biología, Universidad de Murcia, Campus de Espinardo, Murcia 30100, Spain

^2^Departamento de Bioquímica y Biología Molecular-A, Universidad de Murcia, Campus de Espinardo, Murcia 30100, Spain

^3^CNR-IBBR, Istituto di Bioscienze e Biorisorse, UOS di Perugia, Perugia 06128, Italy

* Corresponding authors: José Eduardo Marqués-Gálvez ([joseeduardo.marques@um.es](mailto:joseeduardo.marques@um.es)), Asunción Morte ([amorte@um.es](mailto:amorte@um.es))

^†^These authors contributed equally as senior authors.


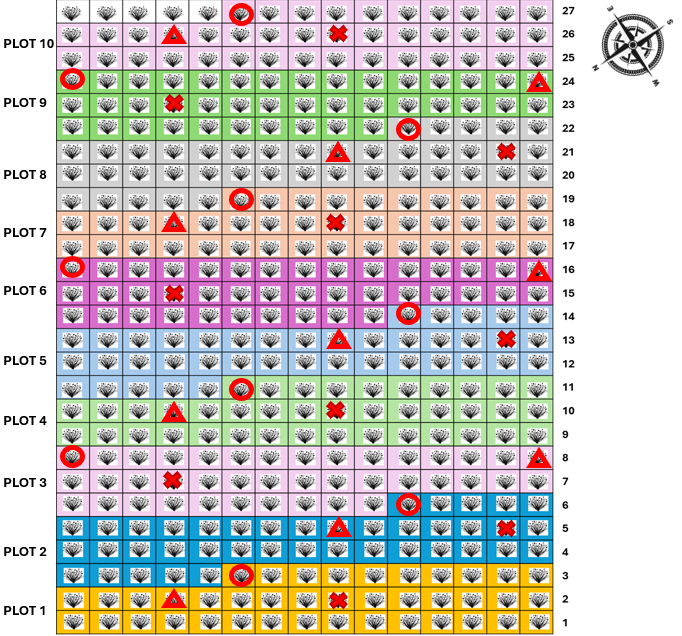


**Supplementary Figure 1.** Graphical representation of the sampling field, showing ten different plots where root and soil samples were collected. Red crosses refers to samples taken in T14, red circles in T18 and red triangles in T27.


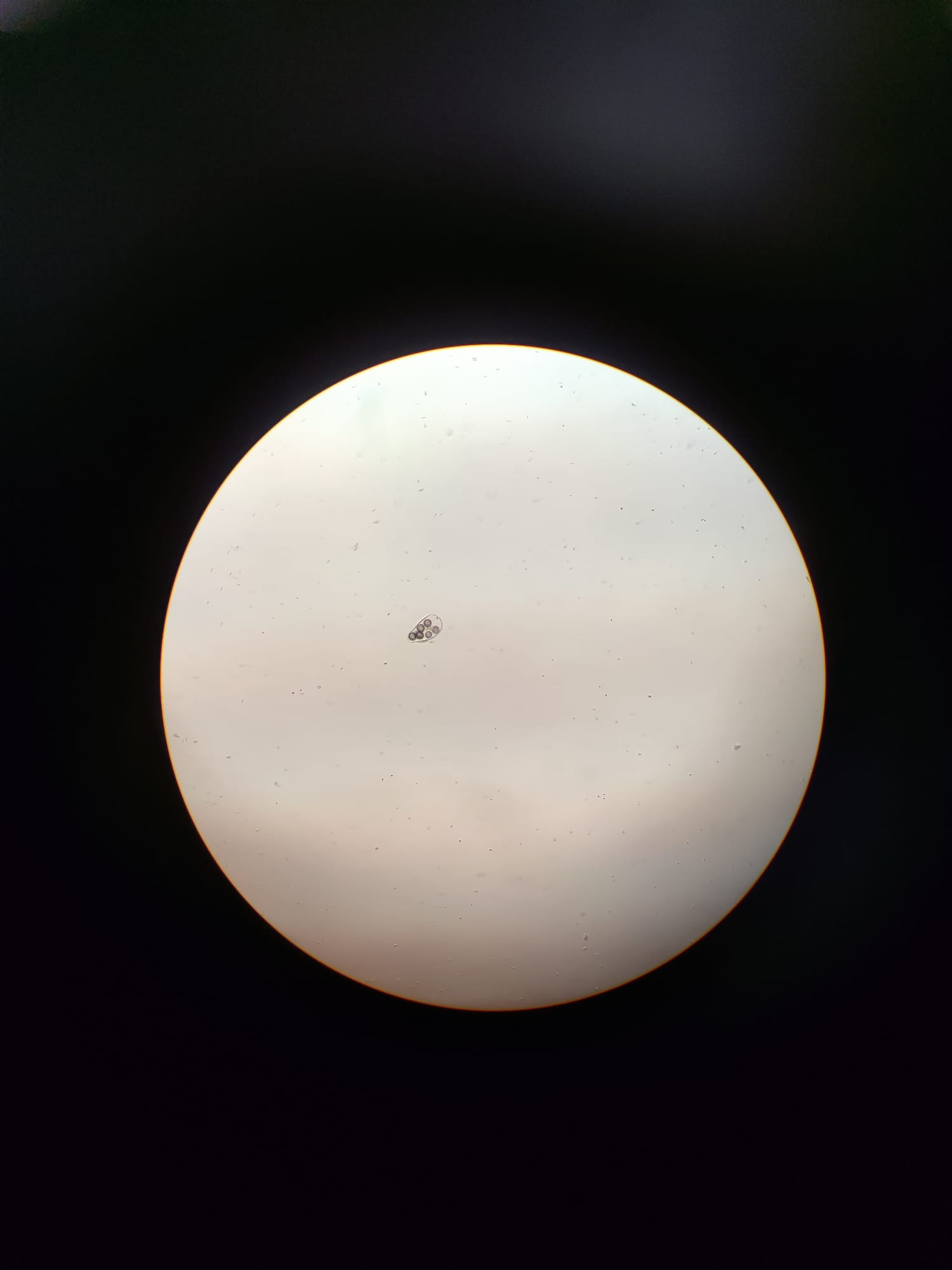


**Supplementary Figure 2.** Spores from a semi-mature gleba with full ornamentation but still inside the asci.


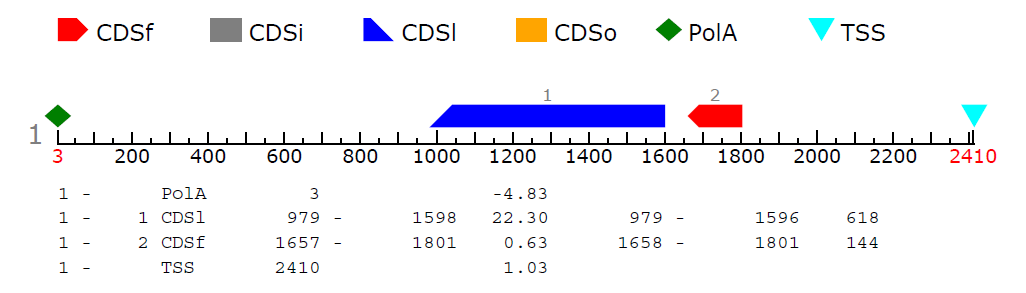


**Supplementary Figure 3.** Prediction of TcMAT1-1 idiomorph based on the Tc1705 amplicon sequence obtained using primers 453-455.


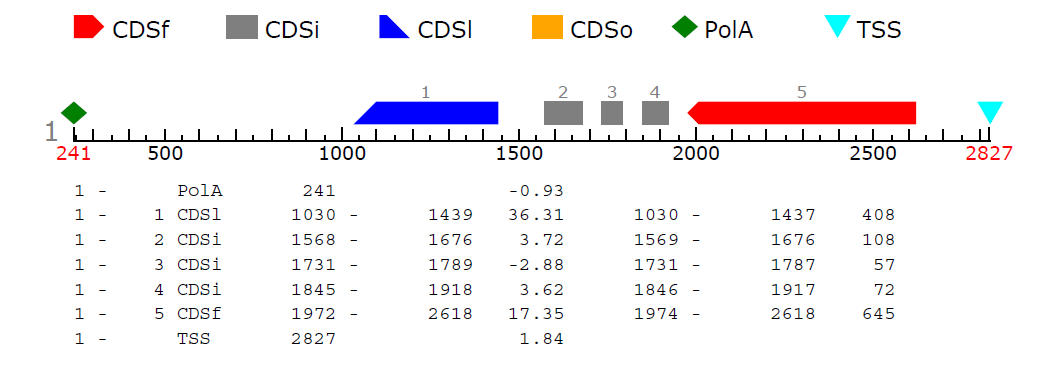


**Supplementary Figure 4.** Prediction of TcMAT1-2 idiomorph based on the TcLlano amplicon sequence obtained using primers 453-455.


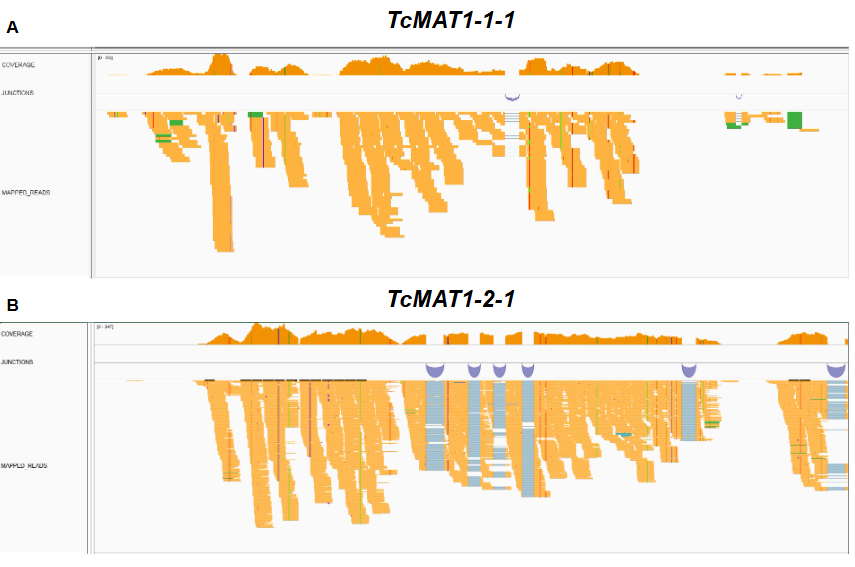


**Supplementary Figure 5.** Visualization of RNA-seq reads from well-watered and drought-stressed *H. almeriense* × *T. claveryi* mycorrhizal roots (SRA ID: SRP272077; Marqués-Gálvez et al. 2021) mapped against the *TcMAT1-1-1* and *TcMAT1-2-1* amplicon sequences.


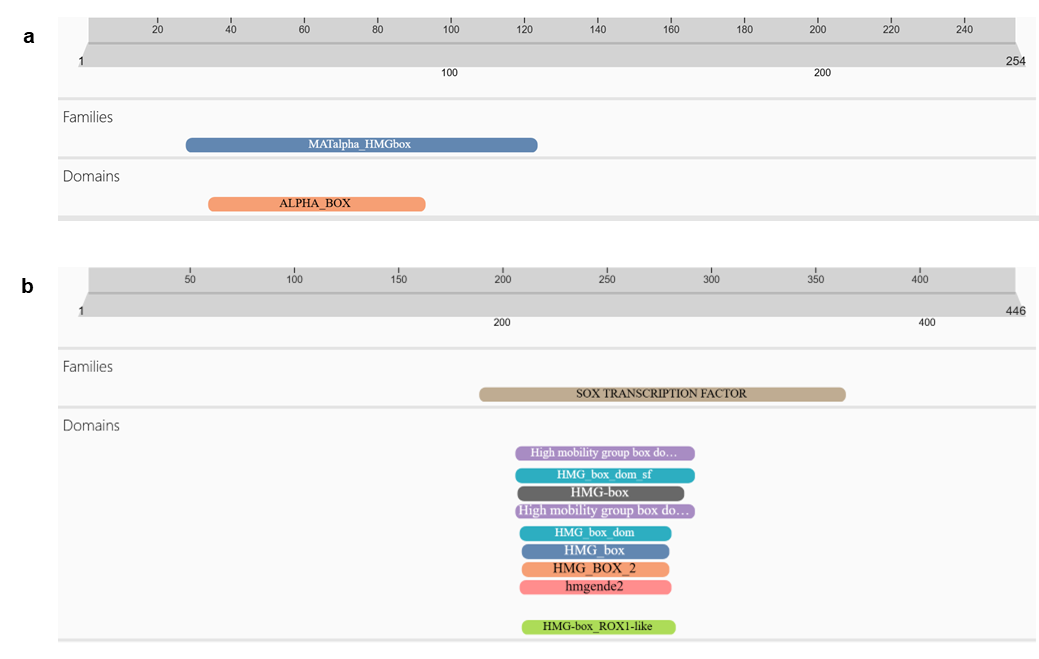


**Supplementary Figure 6.** Protein domain analysis of the TcMAT1-1-1 (a) and TcMAT1-2-1 (b) protein sequences predicted by InterPro.


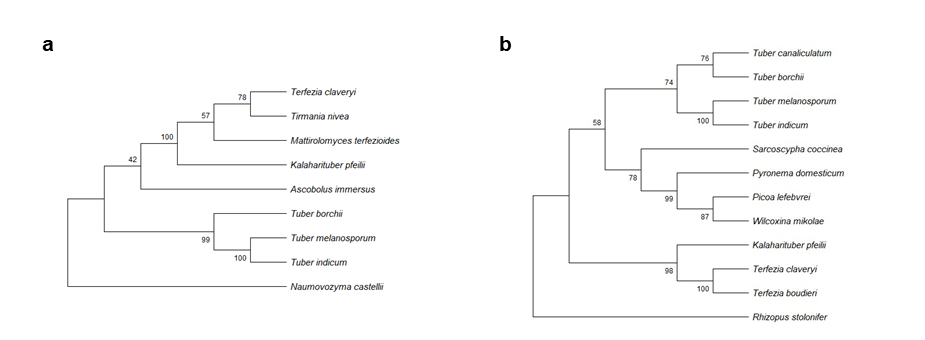


**Supplementary Figure 7.** ITS based phylogenetic trees of the species included in the phylogenetic analyses of the (a) α-box and (b) HMG domains.


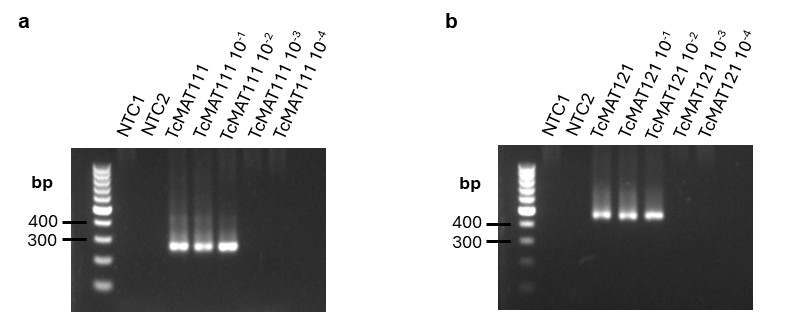


**Supplementary Figure 8.** Agarose gel electrophoresis obtained following the described nested PCR protocol and showing PCR products targeting serial dilutions of: (a) *TcMAT111* (b) *TcMAT121*. Uncropped images can be consulted in Supplementary Dataset 1.
